# Toward whole-genome inference of polygenic scores with fast and memory-efficient algorithms

**DOI:** 10.1016/j.ajhg.2025.05.002

**Published:** 2025-05-26

**Authors:** Shadi Zabad, Chirayu Anant Haryan, Simon Gravel, Sanchit Misra, Yue Li

**Affiliations:** 1 School of Computer Science, McGill University, Montreal, QC, Canada; 2 Parallel Computing Lab, Intel Labs, Bangalore, Karnataka, India; 3 Department of Human Genetics, McGill University, Montreal, QC, Canada

**Keywords:** polygenic risk score, algorithms, linkage disequilibrium, LD, inference, polygenic prediction, whole-genome analysis, GWAS, Bayesian inference

## Abstract

With improved whole-genome sequencing and variant imputation techniques, modern genome-wide association studies (GWASs) have enriched our understanding of the landscape of genetic associations for thousands of disease phenotypes. However, translating the marginal associations for millions of genetic variants to integrated polygenic risk scores (PRSs) that capture their joint effects on the phenotype remains a major challenge. Due to technical and statistical constraints, commonly used PRS methods in this setting either perform heuristic pruning and thresholding or overlook most genetic association signals by restricting inference to small variant sets, such as HapMap3. Here, we present a set of algorithmic improvements and compact data structures that enable scaling summary-statistics-based PRS inference to tens of millions of variants while avoiding numerical instabilities common in such high-dimensional settings. These enhancements consist of highly compressed linkage-disequilibrium (LD) matrix format, which integrates with streamlined and parallel coordinate-ascent updating schemes. When incorporated into our existing PRS method (VIPRS), the proposed algorithms yield over 50-fold reductions in storage requirements and lead to orders-of-magnitude improvements in runtime and memory efficiency. The updated VIPRS software can now perform variational Bayesian regression over 1.1 million HapMap3 variants in under a minute. Using this scalable implementation, we applied VIPRS to 75 of the most heritable, continuous phenotypes in the UK Biobank, leveraging marginal associations for up to 18 million bi-allelic variants. These experiments demonstrated that VIPRS is 1–2 orders of magnitude more efficient than popular baselines while being competitive with the best-performing methods in terms of prediction accuracy.

## Teaser

We present scalable algorithms and data structures for polygenic risk score inference from genome-wide data. Our method enables efficient variational inference on tens of millions of variants, reducing memory and runtime by orders of magnitude and achieving accuracy comparable to state-of-the-art tools.

## Introduction

Polygenic risk scores (PRSs) have recently garnered widespread interest in the clinical research community as a promising tool for patient stratification and personalized medicine.^1^^,^^2^^,^^3^ Polygenic scores quantify the genetic component of complex traits, and their inference can be framed as a high-dimensional multiple regression problem mapping from genotype to the phenotype of interest.^4^ Many methods have been proposed to infer PRSs from large-scale genome-wide association studies (GWASs), each of them tailored to deal with the unique challenges inherent in this form of data (see Pain et al.,^5^ Yang and Zhou,^6^ and Jayasinghe et al.^7^). Because individual-level data are rarely publicly available due to privacy concerns, many PRS inference methods rely on GWAS summary statistics, matched with an appropriate linkage-disequilibrium (LD) reference panel.^5^^,^^6^^,^^8^^,^^9^^,^^10^^,^^11^^,^^12^^,^^13^

While summary-statistics-based PRS methods have been successfully deployed and tested in a variety of biobanks and research settings,^14^ they still face unique challenges of their own. First, these methods often require computing and publicly disseminating large-scale LD matrices from various reference panels to enable users to perform inference on their own GWAS datasets. LD matrices published by popular PRS methods, even when restricted to a relatively small subset of variants and represented in sparse and banded forms, require several gigabytes of storage.^10^^,^^11^^,^^12^^,^^15^ Anything beyond this restricted variant set requires tens of gigabytes to terabytes of storage (cf. Zheng et al.^15^), which makes inference and dissemination difficult. The second challenge concerns heterogeneities between the GWAS summary data and LD reference panels, including differences in allele frequency or LD patterns. Previous work has highlighted the many issues that can arise as a result of these differences, from numerical instabilities to poor prediction accuracy.^13^^,^^15^^,^^16^ Finally, as better variant imputation tools and whole-genome sequencing (WGS) technologies become increasingly integrated into GWAS pipelines, performing PRS inference over millions of variants remains a technical challenge. Commonly used PRS methods usually require hours of runtime and tens of gigabytes of memory for a single phenotype, even when the regression is restricted to a small subset of variants.^6^^,^^15^ Scaling these methods to larger variant sets usually requires expensive specialized hardware.

Here, we present a major methodological update to our previously published work on variational inference of polygenic risk scores (VIPRS)^13^ and provide generally applicable solutions to these challenges. These solutions are summarized in the following four contributions. First, our software tools provide utilities to compute large-scale LD matrices and compress them by over 50-fold. Using our updated LD-matrix storage format, we show that LD matrices for 1.4 million HapMap3+ variants can be shrunk to as little as 300 MB, making them easily shareable via typical workplace communication channels. Second, our study provides concrete recommendations for estimating and constructing well-conditioned LD matrices that behave stably in ultra-high dimensions. Third, we implemented and tested a low-memory version of the coordinate-ascent variational inference (CAVI) algorithm that operates directly on the compressed LD data, which reduces memory footprint by more than an order of magnitude. Finally, we upgraded our software tools to use two layers of parallelism, within coordinate ascent and across independent chromosomes, which provides substantial speedups in inference time up to two orders of magnitude faster than previously published versions of the software.^13^ Putting all of this together makes VIPRS one of the most computationally efficient methods for model-based PRS inference, rivaling even highly optimized implementations of heuristic methods such as clumping and thresholding (C + T).^9^^,^^13^^,^^17^

To demonstrate the promise of these methodological improvements, we applied the latest version of the VIPRS software to 75 of the most heritable phenotypes in the Pan-UK Biobank (Pan-UKB) resource^18^ using all well-imputed bi-allelic genetic variants, reaching up to 18 million variants in European samples. Our analyses demonstrate that the updated variational Bayesian algorithm is able to converge on data of this scale in less than 20 min, using less than 15 GB of RAM. In cross-population and cross-biobank validation analyses, we showed that using dense variant sets may result in small but consistent improvements in prediction accuracy for some models. Given current GWAS sample sizes, the scale of these improvements in accuracy appears to be sensitive to both the statistical and numerical details of the model.

## Material and methods

### Algorithms and data structures for scalable PRS inference

#### Efficient LD matrix storage format and specification

One of the main computational bottlenecks for model-based PRS inference methods is the LD matrix and how it is represented, stored, processed, and ultimately deployed during inference. Recent work has highlighted the potential for orders-of-magnitude reductions in runtime and memory utilization for many routine computational tasks in statistical genetics when the LD matrix (or its inverse) is highly sparse and compactly represented.^19^ As a practical matter, this bottleneck has largely restricted many commonly used PRS methods to a relatively small subset of roughly 1 million HapMap3 variants.^10^^,^^11^^,^^12^ Recent work has explored various strategies and techniques to bypass the difficulty of working with large-scale LD matrices, either by transforming and sparsifying the LD matrix itself^15^^,^^19^ or developing highly scalable algorithms and heuristics that can still work with matrices at the scale of 7–10 million variants.^13^^,^^20^ While the latter approach is useful in principle, it is less scalable, and moving or sharing data of this size can be cumbersome.

**Figure 1:**
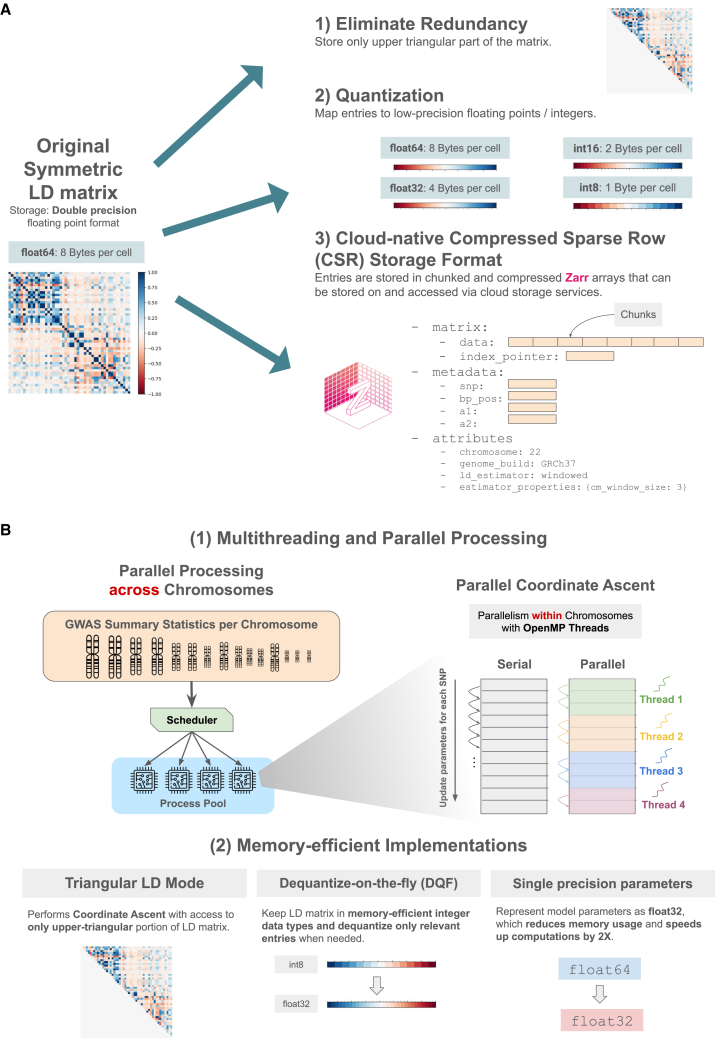
Algorithmic and data-structure improvements in the new VIPRS (A) Three strategies to improve compression, storage, and dissemination of LD matrices. (B) Algorithmic improvements to enhance the efficiency and computational performance of coordinate-ascent inference algorithms.

In this work, we sought to substantially reduce LD storage requirements and its associated costs in terms of memory usage and network traffic. To that end, we took inspiration from LD-matrix storage solutions provided by hail (BlockMatrix)^18^ and the bcor storage format implemented in LDStore.^21^ As summarized in Figure 1, the update encompasses three steps that are simple to implement, fast to run, and generalizable. These updates apply to all LD-matrix estimators outlined in our previous work,^13^ including banded (windowed), block-diagonal, and shrunk LD matrices.

The first step eliminates redundancy and only stores the upper-triangular portion of the symmetric LD matrix (not including the diagonal itself). This step reduces storage requirements by more than a factor of two. The second step involves mapping the entries of the LD matrix to lower-precision floating points or quantizing to integer data types. Previous work has shown that rounding the entries of the LD matrix up to two decimal points has minimal impact on heritability estimation methods.^22^ In the context of fine-mapping, the LDStore software^21^ for computing and storing LD matrices (developed in conjunction with the FINEMAP method^23^) has explored similar compression mechanisms, although documentation of the precise techniques and their impact on inference quality is lacking. We are not aware of similar work in the context of PRS inference. Mapping to lower-precision floating points is as simple as casting the entries of the LD matrix from double-precision floating point (float64) to single-precision floating point (float32) format. This alone can reduce the storage requirements by a factor of 2, going from 8 to 4 bytes per entry. In principle, we can shrink the storage requirements even further by going to half-precision floats (float16). However, we did not explore the half-precision encoding of the data because there is not a universal standard for encoding this data type across programming languages and platforms. Instead, we explored quantizing the data to low-precision integers as an alternative, which allowed us to encode the entries of the LD matrix using a single byte (int8) or two bytes (int16) per entry, representing a reduction of up to a factor of 8 in storage requirements. Table 1 summarizes the implemented data types, their storage requirements, and resolution. Quantization is done using the “scale quantization” technique^24^ whereby the quantized entry $r_{jk}^{\left(q\right)}$ is computed as

$$r_{jk}^{\left(q\right)}=round\left(r_{jk}*s\right)\text{,}$$

where $r_{jk}$ is the Pearson correlation coefficient between variants j and k, the scaling factor $s=2^{b-1}$ is the maximum representable positive value for the integer data type, b is the number of bits used to encode the integer, and round maps the product to the nearest integer. Dequantization reverses the scaling operation: $r_{jk}^{\left(dq\right)}=r_{jk}^{\left(q\right)}/s$.

**Table 1:** Data types for representing the entries of the LD matrix and their properties

**Data type**	**No. of bytes**	**Resolution**
float64	8	$10^{-15}$
float32	4	$10^{-6}$
int16	2	$2^{-15}\approx 0.00003$
int8	1	$2^{-7}\approx 0.008$

Finally, to support the optimized implementations of the coordinate-ascent algorithm in VIPRS, we updated the storage format from variable length (VarLen) arrays to compressed sparse row (CSR) format. In the CSR format, the non-zero entries of the LD matrix are stored contiguously in a very long one-dimensional (1D) array. To delineate the boundaries of each row, we also store an “index-pointer” array that records the start index of each row (Figure 1). These data are stored in a hierarchical, compressed, chunked, and cloud-native storage format known as Zarr (see data and code availability), which supports multi-threaded read and write access. The proposed Zarr storage format represents LD matrices as a hierarchy of arrays, split across two groups. The first group, denoted as matrix, contains the two arrays relevant for the CSR representation: the flattened data array as well as the index pointer (indptr) array. The second group, denoted as metadata, contains arrays of metadata about the variants represented in the matrix, including their rsIDs, genomic coordinates, minor allele frequencies (MAFs), reference and alternative alleles, LD scores, and so forth. Each array is chunked to facilitate efficiently loading and interacting with contiguous subsections of the LD matrix. We used the default chunk size set by the Zarr library (≈1-MB chunks), although fine-tuning this parameter might improve compression or loading speeds. To encourage reproducibility, various “attributes” about the LD matrix may be added to the Zarr hierarchy, including the Biobank from which the matrix was computed, the ancestry of the samples, the sample size, the LD estimator, and its properties. This structure is summarized in Figure 1.

Other features of the Zarr format are also important to highlight in this context. For instance, flexible and powerful compression application programming interface (API) can help shrink the size of the stored data even further. In the latest release, we now use the Zstandard compression algorithm by default instead of the lz4 compressor used in the old format. For LD data, the Zstandard compressor can provide a compression ratio of up to 4 (data not shown). It is also worth emphasizing that Zarr is cloud-native storage format, and this opens the way to provide read access to relevant portions of large-scale LD matrices from cloud storage services directly, without the need to download them locally. Finally, the Zarr storage format is supported through specialized APIs in many popular programming languages, allowing for standardized and universal LD storage specification that can be used for downstream analyses across many statistical genetics toolkits.

#### Optimized and memory-efficient coordinate-ascent algorithms

Most model-based PRS inference methods follow iterative, coordinate-wise sampling or optimization techniques, where the computational cost is dominated by cycling through millions of variants and sampling/updating their parameters conditioned on the parameter values of all the other variants.^8^^,^^11^^,^^12^^,^^13^^,^^25^ In the case of VIPRS, this is represented by the E-step of the variational EM scheme, where we run a coordinate-ascent algorithm to update the variational parameters associated with each variant.^13^ According to our benchmarks, this step can account for up to 80% of the total runtime of the program (data not shown). Thus, optimizing coordinate-wise updates is essential for scaling PRS inference to tens of millions of variants. In the case of VIPRS, these optimizations consisted of five steps.

First, in earlier versions of the VIPRS software (v.0.0.4), we found that the coordinate-ascent step was slowed down due to the VarLen array encoding from Zarr necessitating interaction with the python interface, where loops over millions of elements are relatively inefficient. In the updated software implementation, we rewrote the coordinate-ascent step in pure C/C++, which necessitated using the CSR format for the LD matrix described previously. This update alone resulted in at least 10-fold improvements in speed over the older version of the software (Figure S2).

Second, we optimized the linear algebra operations in the coordinate-ascent updates. If a BLAS library^26^ was detected on the user’s system during installation, we delegated the intensive linear algebra computations to these optimized subroutines for best performance. The manually written linear algebra functions, to which the program reverts if BLAS is not available, were also annotated with compiler hints to use single instruction/multiple data (SIMD) intrinsics where possible, which should enhance their performance considerably.

**Figure 2:**
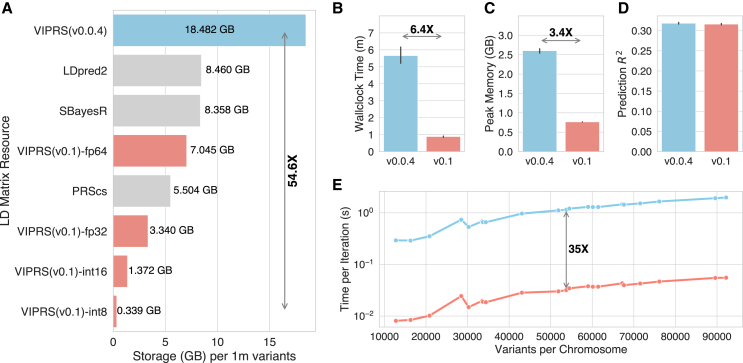
Comparing the computational performance and resource utilization The old (v.0.0.4; sky-blue color) versus newer (v.0.1; salmon color) versions of VIPRS were run with their default settings. All methods were benchmarked on GWAS summary statistics for Standing Height from the UK Biobank, with ≈1.1 million HapMap3 variants included in the analysis. (A) Storage requirements for linkage-disequilibrium (LD) matrices, with matrices from commonly used PRS methods (gray bars) included for comparison. VIPRS v.0.1 supports various data types for storing the entries of the LD matrix, including two float precisions (float64 and float32) and, with quantization, two integer data types (int16 and int8). (B) Total wallclock time (minutes). (C) Peak memory usage (GB). (D) Prediction accuracy (*R*^2^) on held-out test sets in the UK Biobank. (E) Runtime per iteration (in seconds and on log scale) of the two versions of the software as a function of the number of variants on each chromosome. Arrows and bold text in (A)–(C) and (E) indicate the mean fold improvement of the latest version of the software over the old. Vertical black lines above the bars in (B)–(D) show standard errors of each metric across 5-fold of the data.

Third, through profiling, we found that the coordinate-ascent step had become memory bandwidth bound after the above improvements. Therefore, we explored casting all the parameter data types as well as the input data to single-precision floating point (float32) format instead of double precision (float64), which reduced memory bandwidth pressure and runtime per iteration by a factor of two (Figure S2), without significantly affecting prediction accuracy (Figure 2D). The latest version of VIPRS uses single-precision floats by default, although the user has the option to request that the inference be done in double precision.

Fourth, in our previous work, we showed that the variational coordinate-ascent updates can be written so that we access and multiply with the symmetric LD matrix only once per iteration (supplementary note of Zabad et al.^13^; algorithm 1 in Figure S1). In our latest implementation, we provide the option to perform these updates with access to only the upper-triangular portion of the LD matrix (algorithm 2 in Figure S1), thus obviating the need to reconstruct the lower-triangular half and reducing memory usage significantly. To provide an intuition, it helps to think of the original “right-hand updating scheme”^11^^,^^13^ outlined in algorithm 1 (Figure S1) as a message-passing algorithm, where variant j updates its parameters and then broadcasts the information about the change to all its neighbors. In this paradigm, each variant stores information about their neighbors’ posterior mean (weighed by their LD) in the q factor $\left(q_{i}:=\sum_{j\neq i}R_{ij}\eta_{j}\right)$. Under the assumption that the variants are updated in order, it is easy to show that, at any given iteration, it is sufficient for the variant to broadcast the information to variants that come after it. Then, after the coordinate-ascent loop terminates, the information about the changes for variants that come after j can be propagated backward with a simple sparse matrix-vector multiplication with the upper-triangular portion of the LD matrix $R^{\left(UT\right)}$ (last line of algorithm 2 in Figure S1). This “low-memory” or “triangular LD” mode is now the default for the latest version of VIPRS.

Finally, to reduce memory utilization even more, we provide the option for the user to run the software without dequantizing the entire LD matrix beforehand. In this framework, the relevant entries of the matrix are dequantized anew (hence dequantize-on-the-fly or DQF) whenever they are needed during the program’s runtime. This should reduce the memory usage from the LD matrix during inference by up to a factor of 8 (int8 versus float64 representation of LD data), although it comes at a minor cost of extra overhead and redundant computations.

#### Taking advantage of multi-core systems: Parallel coordinate ascent and parallel processing across chromosomes

While the optimizations discussed in the previous section significantly boosted the performance of the software, the algorithm as outlined is still inherently serial and does not take full advantage of modern multi-core computing environments. Other PRS methods already take advantage of parallel processing capabilities in various ways, which enhanced their efficiency considerably.^8^^,^^11^^,^^12^^,^^20^^,^^27^ To make strides in this direction, we identified two areas where parallelism across roughly independent tasks might prove useful for VIPRS.

In the first case, we explored performing inference in parallel across chromosomes, where the GWAS summary statistics and LD reference panel for each chromosome are read and analyzed independently by different system-level processes (Figure 1). This task is done with the help of a process pool interface provided by the joblib python library, where the user can specify the total number of processes that the system should run in parallel. One potential drawback of this approach is that it may result in extra overhead and increased memory usage, depending on the number of processes running concurrently. Nevertheless, it can speed up runtime significantly and it can be accessed by simply setting the flag –n-jobs when running our updated command-line interface (CLI) script viprs_fit (see data and code availability).

In the second case, we explored performing parallel updates within the coordinate-ascent algorithm itself by leveraging the OpenMP multi-threading API. Parallel coordinate-ascent algorithms have been studied extensively in the literature, especially with regard to their convergence properties.^28^ In VIPRS, the implementation of this scheme is as follows. Instead of updating the parameters of the variants serially as outlined in algorithms 1 and 2 (Figure S1), we divide the variants into chunks and update these chunks simultaneously and in parallel with OpenMP threads (Figure 1). In idealized settings, this should speed up both triangular and symmetric LD algorithms by a factor proportional to the number of threads. However, in practice, there may be some overhead, especially for smaller chromosomes where the runtime per iteration is already negligible. An important detail to highlight here is that, unlike some previous implementations (e.g., Lassosum^8^), our parallel coordinate-ascent algorithm is not restricted to LD blocks and works seamlessly with any LD-matrix structure (windowed, shrunk, or block).

We caution users regarding multi-threading in the coordinate-ascent step paired with triangular LD as in algorithm 2 (Figure S1). In our experiments, we noticed that this setup, when coupled with a small variants/threads ratio, can result in some instabilities and oscillations in the parameter values. This is due to “staleness” of the parameters (e.g., q factor is one iteration behind), and such effects have been documented in the literature on parallel and asynchronous coordinate ascent.^29^^,^^30^ To resolve this issue, we implemented a heuristic that decreases the number of threads by one when instabilities are detected. In future work, introducing a step size (or learning rate) as suggested by Liu et al.^30^ may be a more appropriate and general solution. In addition, staleness and oscillations can make the convergence slower, which necessitates running the EM algorithm for more iterations, hence counteracting the benefits of multi-threading in these cases.

#### Fast and accurate hyperparameter tuning

In previous work, we demonstrated that tuning some of the hyperparameters (e.g., fraction of causal variants) of the VIPRS model via cross-validation and grid search improves out-of-sample prediction accuracy compared to the standard variational expectation maximization (VEM) algorithm.^13^ The previous grid-search implementation, however, was hampered by two main limitations. First, cross-validation required access to held-out validation data in the form of individual-level genotype and phenotype data or validation summary statistics, which may not be available in some scenarios. Second, the previous grid-search implementation, VIPRS-GS, was notably three times slower and required a lot more computational resources than the simple VEM variant of the algorithm.^13^ To address these limitations, the latest version of the VIPRS software (v.0.1.3) provides two features that enable scaling cross-validation and hyperparameter tuning to millions of genetic markers.

First, to obviate the need for external validation data, our software package magenpy v.0.1.5 now provides interfaces and utilities that implement the PUMAS procedure of Zhao et al.,^31^ which produces GWAS summary statistics for training and validation sets, conditional on the original GWAS marginal effects and LD matrix. This is done by specifying the proportion of samples to use for training, $p_{\text{train}}$ and then sampling the marginal effect sizes of the training subset conditional on the LD matrix^15^^,^^31^:

$$\begin{array}{l} {\hat{\beta }}_{\text{train}}∼N\left({\hat{\beta }}_{\text{GWA}},\left(\frac{1}{Np_{\text{train}}}-\frac{1}{N}\right)R\right)\\ {\hat{\beta }}_{\text{validation}}=\frac{1}{1-p_{\text{train}}}{\hat{\beta }}_{\text{GWA}}-\frac{p_{\text{train}}}{1-p_{\text{train}}}{\hat{\beta }}_{\text{train}} \end{array}\text{.}$$

Here, ${\hat{\beta }}_{\text{GWA}}$ is the marginal GWAS effect size for the entire sample, N is the GWAS sample size, and ${\hat{\beta }}_{\text{train}}$/ ${\hat{\beta }}_{\text{validation}}$ are the marginal effect sizes for the training and validation subsets, respectively. Sampling from multivariate Gaussian distributions conditional on high-dimensional LD matrices is computationally prohibitive, thus we resort to approximations as discussed in previous work.^15^^,^^31^ In our implementation, we split the matrix into contiguous sub-blocks, where no block is larger than a user-specified threshold. By default, we set the maximum block size to 500 variants, as we observed empirically that it maintains good balance between accuracy and efficiency. Sampling for each sub-block is done via numpy’s multivariate_normal random generator^32^ with the factorization method set to eigen decomposition (eigh) by default. To scale this procedure to matrices with millions of variants, our software also supports parallel processing across blocks with thread pools. When combined with our chunked and compressed LD-matrix format, this implementation of the PUMAS sampling procedure requires only several minutes of runtime and modest memory resources for 18 million variants.

Second, while our improved parallel coordinate-ascent algorithms made great strides in addressing the second limitation of grid search (see Figure S4), performing inference even with modestly sized grids can still be time consuming in high dimensions. To overcome this, we implemented a pathwise grid-search algorithm for VIPRS, inspired by the well-known pathwise algorithm for the LASSO.^33^ Instead of fitting model parameters separately for each grid point, the pathwise algorithm sorts the hyperparameters in the grid (e.g., the proportion of causal variants π) in ascending order and leverages parameter estimates from prior solutions as warm-start initialization for the current grid point. In practice, we found that this simple procedure reduces the total number of iterations and inference time by ≈30% while maintaining the same prediction accuracy as an independent grid search (Figure S17).

#### General software usability improvements

The algorithmic improvements described earlier would be inaccessible without software design and tools that are easy to distribute, in addition to being well tested and documented. Since their last release, our python packages magenpy (our in-house toolkit for GWAS and LD data harmonization and simulation) and viprs (see data and code availability) have undergone extensive refactoring to simplify the code, add basic unit and integration tests, make it more robust, and document most of the modules, functionalities, and interfaces. Specifically, we now follow a continuous integration/continuous development (CI/CD) testing regime whereby any updates to the software have to pass basic functionality testing across different platforms, including Windows, MacOS, and Linux. Furthermore, both packages now have dedicated web pages with documentation that includes installation guidelines, walk-throughs of the basic features, basic tutorials, and API references. These resources are not yet complete, and we anticipate that they will grow further in the future, especially as they gain interest from the research community. Additionally, there remain some challenges with distributing the software across platforms, given the wide range of dependencies that the packages require. To facilitate distribution and widespread adoption, we also now provide Docker scripts^34^ that containerize both magenpy and viprs in addition to all their essential dependencies. Both packages are open source and distributed under the MIT license.

### Improving numerical stability of sumstats-based PRS methods

#### Causes of numerical instabilities in PRS inference

In previous work, we noted that VIPRS and other summary-statistics-based PRS methods are prone to numerical instabilities in some settings.^13^ These numerical issues are characterized by the parameter estimates β exploding in magnitude and the mean squared error (MSE) component of the objective function becoming negative. We initially hypothesized that these issues were partly caused by heterogeneities between the GWAS summary statistics and the LD reference panel and recommended performing quality control (QC) filtering using tools such as DENTIST^35^ before fitting the model. This explanation, however, was incomplete, as these numerical issues have been subsequently detected even in cases where the GWAS summary statistics and the LD panel originate from the same cohort.

Here, we propose that another main cause of these numerical pathologies can be traced back to the spectral properties of the LD matrix itself. Model-based PRS methods typically aim to optimize or approximate an objective that includes the MSE, a quantity that can be expressed in terms of the marginal effect sizes $\left({\hat{\beta }}_{\text{GWA}}\right)$ and the in-sample LD matrix (R):

$$\begin{array}{ll} MSE\left(\beta \right)\equiv \frac{1}{N}\|y-X\beta {\|}_{2}^{2} & =\frac{1}{N}\left[y^{⊤}y-2y^{⊤}X\beta +\beta^{⊤}X^{⊤}X\beta \right]\geq 0\\ & =1-2{\hat{\beta }}_{\text{GWA}}^{⊤}\beta +\beta^{⊤}R\beta \geq 0 \end{array}\text{.}$$

The equality holds under the assumptions that both the genotype matrix X and the phenotype vector y are fully observed, noise free, and standardized column-wise. In practice, X can contain substantial amounts of missing values and noisy entries due to variant calling or imputation errors. Furthermore, even if X is perfectly observed, the entries of R are estimated in a pairwise fashion and then further shrunk, thresholded, and compressed. All of these sources of noise can make our approximation of the in-sample LD, denoted as $\tilde{R}$, not positive semi-definite (PSD), which can lead the quadratic form $\beta^{⊤}\;\tilde{R}\beta$ to become negative. To be precise, if the minimum eigenvalue of the approximate LD matrix $\lambda_{\min}\left(\tilde{R}\right)$ is strongly negative, the optimization for β can run away toward the corresponding eigenvector and create negative errors, leading directly to the numerical explosions reported in the PRS literature.^13^^,^^15^

Aside from the spectral properties of the approximate LD matrix $\tilde{R}$, another problem that can lead to numerical instabilities is over-sparsifying or shrinking the LD matrix or substantial heterogeneities between the summary statistics and the LD reference panel.^13^^,^^15^^,^^16^ This issue becomes especially prominent when the model requires estimating the residual variance $\sigma_{\varepsilon }^{2}$. To take an extreme example, assume that we approximate R with the identity matrix I. While this choice is guaranteed to be PSD, it could nonetheless result in numerical instabilities because it will largely underestimate the quadratic form $\beta^{⊤}R\beta$, which again can result in a negative MSE in some circumstances. This issue is harder to diagnose and satisfactorily address in practice, except via discarding heterogeneous summary data.^16^^,^^35^

#### Analyzing the spectral properties of LD matrices

To facilitate analyzing the spectral properties of LD matrices, we developed data structures and dedicated software tools for computing extremal eigenvalues of LD matrices efficiently. Our software package magenpy now provides a linear operator class,^36^ called LDLinearOperator, which enables users to perform a wide range of linear algebra operations on LD matrices in their compressed form (i.e., while they remain triangular and quantized). To rapidly estimate the extremal eigenvalues of an LD matrix, we use iterative methods provided by scipy sparse matrix interfaces for the ARPACK library.^36^^,^^37^ These routines can estimate the minimum eigenvalue of chromosome-wide LD matrices with hundreds of thousands of variants in a matter of minutes, using limited memory footprint.

Our analyses indicate that many common approaches for estimating LD matrices in the context of PRS inference result in non-PSD matrices with large negative eigenvalues. Specifically, there are three main contributing factors that can impact the spectrum of estimated LD matrices: sparsification pattern, pairwise correlation estimator in the presence of missing data, and approximation error. Sparsification pattern is the manner by which the sample LD matrix is transformed into a sparse one, using, e.g., banded or block-diagonal masks. Our analyses indicate that banded LD matrices with fixed window size, e.g., 3 centimorgans (cM), tend to have relatively large negative eigenvalues, especially when the sample size of the reference panel is small and/or in the presence of variants in long-range LD (LRLD) regions (Figure S8). Block diagonal matrices are supposed to be more well conditioned in principle, since each block is a Gram matrix by construction, and the eigenvalues of the entire LD matrix is simply the concatenation of the eigenvalues of individual blocks.^38^ This observation explains why block-diagonal matrices are popular in PRS inference methods,^8^^,^^10^ especially methods that are applied to larger variant sets.^15^

However, even if block-diagonal LD matrices are used in practice, the resulting matrices may still have large negative eigenvalues because of the other two contributing factors. First, many pairwise correlation estimators from genotype data, such as plink1.9,^17^ use an unbiased estimator of Pearson correlation that discards missing observations. As a result, the entries of the LD matrix are estimated independently using disjoint subsets of the data. Thus, we expect that in the presence of missing genotype calls due to hard thresholding, this will affect the spectra of estimated LD matrices.^39^ Our analyses indicate that this indeed can result in matrices with large negative eigenvalues, even when using block-diagonal masks (Figure S9). Instead of discarding missing genotypes, an alternative is to impute them using, e.g., mean imputation (MI).^39^ This approach produces LD matrices that are nearly positive semi-definite and have a more stable spectrum (Figure S9). For a detailed discussion of the benefits and drawbacks of both approaches, we refer the reader to the work of Choi and Tibshirani.^39^

Finally, other sources of approximation error can also impact the spectra of estimated LD matrices. For example, thresholding individual entries or using ultra low-precision storage formats (e.g., quantization to int8) will negatively affect the spectrum, especially for large-scale LD matrices (Figure S9). Therefore, there is a trade-off to be struck between compressibility and producing well-conditioned matrices.

#### Proposed solutions and recommendations to achieve numerical stability

Although objective functions of the form discussed above have been carefully explored in the statistics literature over the past decade,^39^^,^^40^^,^^41^ it remains scarcely cited in statistical genetics, with some exceptions (e.g., Escribe et al.^42^). In particular, the pioneering work of Loh and Wainwright^40^ introduced losses of this form to allow for fitting penalized regression models when the design matrix X contains missing or noisy features. The authors noted the potential pitfalls with non-PSD covariance matrices and recommended constraining the β parameters during optimization to restore the convexity of the LASSO objective and to prevent numerical instabilities. In that original work, the authors proposed a projected gradient descent algorithm to perform this constrained minimization, which can be impractical in high-dimensional settings. An alternative approach was proposed by Datta and Zou,^41^ where the authors advocated projecting $\tilde{R}$ to the nearest PSD matrix and then using efficient coordinate-ascent algorithms to optimize the modified objective. This approach is promising in principle, although projecting large-scale LD matrices onto the PSD cone can be prohibitively expensive and may destroy the sparsity structure of $\tilde{R}$. One way to bypass this difficulty is to work with block-diagonal LD matrices, whereby each block can be projected, denoised, and post-processed separately, as done in two recent PRS methods^15^^,^^43^ as well as in the software implementation of PRS-CS.^10^ However, previous work indicated that banded LD matrices may perform slightly better than block-diagonal ones in some settings,^13^^,^^16^ which motivated us to explore more general solutions.

Here, we followed a third approach outlined by Choi and Tibshirani,^39^ where instead of using the approximate LD matrix $\tilde{R}$ in the objective, we use $\tilde{R}+\left|\lambda_{\min}\right|I$, where $\left|\lambda_{\min}\right|$ is the absolute value of the smallest (negative) eigenvalue of $\tilde{R}$ and I is the identity matrix. This is similar in principle to the approach outlined by the developers of the Lassosum2^16^ method, although in that work the authors perform uninformed grid search over the hyperparameter instead of deriving it from the spectral properties of the LD matrix itself. In the case of VIPRS, this simple modification affects the evidence lower bound (ELBO),^13^ which includes the expectation of the MSE with respect to the variational density $E_{q}\|y-X\beta {\|}_{2}^{2}$. Building on our previous work^13^ and replacing $\tilde{R}$ with $\tilde{R}+\left|\lambda_{\min}\right|I$, this expectation evaluates to

$$N\left(1-2{\hat{\beta }}^{⊤}\eta +\eta^{⊤}\tilde{R}\eta +\left(1+\left|\lambda_{\min}\right|\right)\sum_{j=1}^{M}Var_{q}\beta_{j}\right))\text{.}$$

This, in turn, affects the updates for the variational parameters, mainly through the posterior variance estimate $\sigma_{jk}^{2}$:

$$\sigma_{jk}^{2}=\frac{\sigma_{\varepsilon }^{2}}{N\left(1+\left|\lambda_{\min}\right|\right)+\frac{\sigma_{\varepsilon }^{2}}{\sigma_{k}^{2}}}\text{.}$$

Thus, the main effect of adding the $\left|\lambda_{\min}\right|$ to the objective is that it constrains the posterior for the effect size and acts as an additional shrinkage penalty.^16^^,^^39^ The strength of this penalty is proportional to the magnitude of the smallest negative eigenvalue: The more ill-conditioned the LD matrix, the stronger the penalty. In practice, when working with large-scale banded LD matrices, we found that this can result in overshrunk effect-size estimates, which may lower prediction accuracy.

To deal with the potential for overshrinkage and underfitting, we provide two alternative solutions. In the first case, if an independent validation dataset is available, we allow the user to explore an informed grid over multiples of the parameter $\left|\lambda_{\min}\right|$, e.g., the grid can be constructed as: $\left\{0,0.01,0.1,1,2\right\}*\left|\lambda_{\min}\right|$.^16^^,^^39^ We then choose the penalty level that maximizes prediction on the held-out validation set. In the second case, instead of using a single $\left|\lambda_{\min}\right|$ penalty chromosome wide, we allow the penalty to vary over genomic blocks. This is inspired by our observation that some genomic regions (e.g., long-range LD) contribute differentially to the negative eigenvalues of the LD matrix (Figure S8), and penalizing variants in those problematic regions more strongly will stabilize the optimization algorithm without overshrinking variants in more well-behaved regions.

In summary, our recommendations for constructing large-scale and well-conditioned LD matrices are as follows. In general, we recommend using block-diagonal masks for sparsifying the matrix. If the genotype matrix contains missing genotype calls, it is advisable to perform either MI or estimate pairwise correlation based on raw dosages. To reduce approximation errors, it may be beneficial to use int16 data type for storage, although in our experiments we found that int8 works fine in most cases. If any of these conditions are unattainable for technical or other reasons, we recommend using the $\left|\lambda_{\min}\right|$ penalty approach to constrain the parameter estimates and stabilize the inference process.

### Benchmarking experiments

#### Data used for benchmarking

To assess the impact of the algorithmic modifications introduced in the latest version of VIPRS, we used a subset of the summary statistics and LD reference data that was generated for the original VIPRS paper.^13^ For the LD reference panel, we used LD matrices for approximately 1.1 million HapMap3 variants, computed using the windowed estimator with a window size of 3 cM (see data and code availability). These old LD matrices were converted to the proposed CSR format using a custom script (see data and code availability), and the entries were cast and/or quantized from the standard double-precision float (float64) to the four different data types listed in Table 1.

As for the GWAS summary statistics, we used the same summary data for standing height that was generated in our previous study.^13^ In that analysis, we performed 5-fold cross-validation on the White British subset in the UK Biobank (N=337,225), where, for each fold, 80% of the samples were used for computing GWAS summary statistics and the remaining 20% were used as a held-out test set. Thus, our main benchmarking results are shown as averages and standard errors across the five independent replicates of summary statistics for standing height in the UK Biobank.^44^

#### Benchmarking setup, metrics, and configurations

Our benchmarking experiments were focused on assessing the performance and resource usage of the VIPRS software along four axes. The first axis is the total wallclock time, which measures the time it takes to read the summary statistics files from disk, perform variant matching between the summary data and LD metadata, load the relevant entries of the LD matrix to memory, and perform variational inference to approximate the posterior for the effect sizes. Our optimizations should decrease the total wallclock time in three ways: (1) reading the LD matrix from disk should be significantly faster, since stored entries are highly compressed; (2) the coordinate-ascent updates should take considerably less time; and (3) parallelism, both within a coordinate-ascent step and across chromosomes, should also reduce the total wallclock time. The second axis of performance is peak memory used throughout the program. This metric was measured using the ∖usr∖bin∖time command on the Linux cluster where the experiments were done.

These first two measures are dependent on the characteristics of the data, convergence criteria, and the amount of computational resources used, which motivates the third axis: the runtime per iteration. This metric measures the time it takes to complete a single coordinate-ascent step (also called the E-step in our previous work^13^). Benchmarking the coordinate-ascent step is done with a custom script (see data and code availability) where, after initializing the VIPRS module with data for a given chromosome, we invoke the coordinate-ascent function K times to reach a total runtime of roughly 1 s. From this, we estimate the runtime per iteration. To obtain robust confidence intervals, we repeat this operation a total of 15 times.

The fourth and final axis is prediction accuracy for standing height on the held-out test set. Prediction accuracy was measured using the pseudo *R*-squared metric as implemented in the viprs package.^13^ The metric requires access to GWAS summary statistics from the held-out test set of each fold.

Users of the software can also assess some of these axes of performance on their own personal computing environments, by specifying the –output-profiler-metrics flag to the CLI script viprs_fit.

### Large-scale PRS analyses with Pan-UK Biobank GWAS data

#### Selection criteria for phenotypes from the Pan-UKB GWAS resource

The Pan-UKB is a comprehensive study that aims to elucidate the genetics of thousands of measured phenotypes from the UK Biobank across all ancestry groups.^18^ GWAS summary statistics are available for 7,221 phenotypes across six continental ancestry groups, in addition to LD score (LDSC) heritability estimates and other QC flags^18^ (see data and code availability). For our analysis, we selected continuous phenotypes that passed all the QC criteria defined by the Pan-UKB study in at least three different ancestry groups. These QC criteria included sample-size checks and restricting to phenotypes with bounded LDSC heritability estimates that are significantly greater than zero. Applying these filters results in 75 high-quality and heritable phenotypes that span seven different categories, from blood biochemistry to anthropometric measures. The phenotypes and their codes, categories, description, and heritability estimates are listed in Table S1.

#### Extracting UK Biobank genotype and phenotype data

If polygenic scores are to be useful in clinical settings, they must be applicable to individuals of different ancestries. Toward this goal, we expanded the scope of our analyses and the pre-computed LD reference panels that we provide to encompass six ancestry groups, previously defined by the Pan-UKB project^18^ (Return 2442 from the UK Biobank dataset^44^). Specifically, Return 2442 provides principal=component analysis (PCA) coordinates and covariates data for 448,216 diverse samples in the UK Biobank, in addition to a column assigning each sample to one of the following six continental ancestry groups: AFR (African), AMR (Admixed American), CSA (Central and South Asian), EAS (East Asian), EUR (European), and MID (Middle Eastern). Out of these samples, we selected individuals who have no close relatives in the dataset (based on the related column provided), which reduced the final sample size to N=382,329 individuals. The breakdown of sample sizes by ancestry is shown in Table 2.

**Table 2:** Sample sizes of unrelated individuals in the UK Biobank across the six ancestry groups defined by the Pan-UKB project

**Ancestry group**	**Code**	**Sample size**
African	AFR	6,255
Admixed American	AMR	987
Central and South Asian	CSA	8,284
East Asian	EAS	2,700
European	EUR	362,446
Middle Eastern	MID	1,567
All samples	ALL	382,239

We followed a similar variant quality control protocol as the Pan-UKB study.^18^ Specifically, we selected bi-allelic variants with INFO score ≥0.8 and minor allele count (MAC) of at least 20 across the entire cohort. The only additional filter that we applied is excluding variants with ambiguous strand. This resulted in a total of 23,332,104 variants that were used in our largest downstream analysis.

To generate smaller subsets of variants for more computationally tractable workflows, we applied two additional filters. In the first case, we applied a minor allele frequency filter of MAF ≥0.1%, which resulted in a dataset of 13,772,714 variants. In the second case, in addition to the MAF filter, we restricted to the expanded HapMap3+ set generated by the authors of the LDPred2 software,^12^^,^^45^ which resulted in a dataset of 1,431,634 variants.

With these filters at hand, we used plink2 v.2.00a6LM^17^ to extract and transform the UK Biobank genotype dosage data from the provided bgen files^44^ to plink1 BED file format, with a hard-call threshold of 0.1. We also used plink1.9 v.1.90b4.6 to annotate the extracted bim files with the centimorgan distance using the HapMap3 genetic map.^46^

To evaluate the predictive performance of the inferred PRS models, we also extracted phenotype measurements for the 75 phenotypes selected earlier (Table S1). The phenotypes were extracted from the tabular data of the UK Biobank resource and for all participants. If multiple measurements for the same phenotype were available (due to repeat visits), we selected the initial measurement. For the purposes of evaluation, the phenotypes were kept on their original scale. The only QC filter that we applied for each phenotype was removing outliers, defined as measurements exceeding 3 standard deviations from the mean.

#### Computing LD matrices for all Pan-UKB populations

With the extracted UK Biobank genotype data, we then used our software package magenpy to compute LD matrices for the six ancestry groups listed in Table 2. Within each ancestry group, we restricted data to variants with MAC of at least 20. To explore the spectral properties of LD matrices and their impact on numerical stability and inference quality, we computed LD matrices using three different settings.

First, we computed LD matrices using both windowed (i.e., banded) and block-diagonal masks. Windowed masks estimate LD between all pairs of variants that are at most 3 cM apart on the same chromosome. Block-diagonal masks compute LD between all variants within the same LD block, defined by the LDetect software^47^ (see data and code availability). The LDetect authors only published block-diagonal masks for African, Asian, and European samples, broadly defined.^47^ Following previous work in this space,^10^ we used the European block boundaries for EUR and MID ancestry groups, African boundaries for the AFR and AMR ancestry groups, and Asian boundaries for the EAS and CSA ancestries.

Second, we estimated LD matrices using two strategies for dealing with missing genotype calls: MI or simply discarding missing observations, as done in the LD estimators provided by plink1.9 v.1.90b4.6.^17^ Third, to examine the impact of quantization on compressibility and the spectral properties of LD matrices, we stored the data using both int8 and int16 quantization. In the main analyses of the paper, we mainly used block-diagonal LD matrices that are computed with MI for missing values and stored using int8 quantization.

Due to the relatively small sample sizes of the minority populations, we only computed their LD matrices using the HapMap3+ subset of variants. For the European samples, we computed three sets of LD matrices corresponding to each of the variant sets described above. The largest variant set (INFO >0.8) yielded pairwise correlations between 17,745,575 variants in European samples, with the reduction mainly due to the threshold MAC being >20. The number of variants and storage size of each of the LD matrices is shown in Table 3. While the variant sets overlap with one another, due to the size of the larger matrices, we created separate copies for each variant set. The HapMap3+ LD matrices for the six ancestry groups are available for download via GitHub and Zenodo (see data and code availability).

**Table 3:** LD-matrix storage size for each ancestry group in the Pan-UKB, containing pairwise correlations between well-imputed variants (INFO >0.8)

**Ancestry group**	**Variant set**	**No. of variants**	**LD-matrix storage size (GB)**	
			**int16**	**int8**
AFR	HapMap3+	1,259,175	0.59	0.23
AMR	HapMap3+	1,286,943	0.63	0.26
CSA	HapMap3+	1,379,991	1.12	0.36
EAS	HapMap3+	1,146,910	0.79	0.27
MID	HapMap3+	1,307,269	0.94	0.36
EUR	HapMap3+	1,431,634	1.01	0.31
EUR	MAF >0.1%	13,492,321	70.72	13.79
EUR	MAC >20	17,745,575	118.91	20.66

For European samples, LD-matrix storage size is shown across three different variant sets. LD matrices were estimated using block-diagonal masks from LDetect (see data and code availability) and stored using int8 and int16 quantization.

#### Training VIPRS on Pan-UKB GWAS summary statistics

We split the Pan-UKB GWAS summary statistics files for the 75 continuous phenotypes per ancestry and merged them with variant and phenotype metadata provided in the Pan-UKB manifest (see data and code availability). Some phenotypes did not have GWAS summary statistics for some ancestry groups, due to limited sample sizes or other QC criteria.

After the summary statistics files were merged and post-processed, we fit VIPRS v.0.1.2 on all the available summary data, each of which was matched with the appropriate ancestry-specific LD reference panel described earlier. To reduce memory usage, inference was carried out with the triangular LD mode and with the dequantize-on-the-fly flag, which dequantizes the entries of the LD matrix only when needed. By default, the coordinate-ascent inference step was done using four threads for the HapMap3+ reference panel, and eight threads were used for the 13m (MAF >0.001) and 18m (MAC >20) reference panels.

For the grid search version of the algorithm (VIPRS-GS), we used the PUMAS procedure^31^ (available as of v.0.1.3) (see “fast and accurate hyperparameter tuning”) to sample training and validation summary statistics, with the training proportion set to 0.8 by default. The search was over the hyperparameter π (proportion of causal variants), and the grid consisted of 20 points on a $\log_{10}$ scale from (10/M) to 0.2, where M is the number of variants. Once all the models along the grid had converged, we used the validation pseudo-$R^{2}$ metric^8^^,^^13^^,^^15^ to select the best-performing model. We then refitted the VIPRS model on the original GWAS data with the hyperparameter π fixed to the value selected in the grid search.

#### Training baseline methods on Pan-UKB GWAS summary statistics

To place the statistical and computational performance of the updated VIPRS software in context, we performed inference on the Pan-UKB GWAS summary statistics using two commonly used PRS methods: LDpred2^12^ and SBayesRC.^15^ A comprehensive comparison with six established PRS methods can be found in our previous work.^13^

For the LDpred2 software, since there are no validation data, we used the auto version of the method, as implemented in bigsnpr v.1.12.18 (see data and code availability), leveraging the HapMap3+ LD reference panel published by the authors (see data and code availability). LDpred2-auto performs inference over a grid of 30 prior probabilities, extending from $10^{-4}$ to 0.2 on a log scale. Once the Markov chain Monte Carlo chains have terminated, they are filtered to remove outlier chains (outside the 95% percentile) and averaged to obtain the posterior mean estimates for the effect sizes. As recommended by the authors,^12^ we ran the software genome wide across all chromosomes.

While both LDpred2 and SBayesRC employ Gibbs sampling to approximate the posterior for the effect sizes, SBayesRC adopts a fully Bayesian approach, assigns priors to most of the hyperparameters of the models, and approximates their posterior distribution accordingly. Additionally, SBayesRC makes use of a low-rank representation of the LD matrix,^15^ which enabled scaling it to 7 million variants. In our pipeline, we used version v.0.2.6 of the SBayesRC software (see data and code availability), distributed via a Docker image provided by the authors. For the Pan-UKB analyses, we ran the method without annotations, using both the HapMap3 variant set as well as the 7 million variant set (see data and code availability).

On the computational resource side, all methods were allocated ten cores and ran with eight threads, similar to the VIPRS models described above.

#### Cross-ancestry evaluation of inferred PRS models in the UK Biobank

Our previous work established that VIPRS is competitive with state-of-the-art PRS methods in terms of prediction accuracy and that relative performance of PRS models within the training cohort tends to correlate with their relative accuracy in other ancestries.^13^ In this study, we focused on cross-population transferability to assess prediction accuracy within the UK Biobank. Given the six ancestry groups defined by the Pan-UKB, we trained a PRS model on GWAS summary statistics from one reference population and then evaluated its prediction accuracy on the remaining five populations. In addition to helping assess transferability, this procedure ensures that the test set is distinct from the training set. Also, unlike cross-biobank evaluation, this procedure is less prone to batch effects in genotyping technology or phenotypic measurements, since all of the data were collected and preprocessed in the same manner. One potential limitation of this procedure is that cross-population transferability could be affected by many factors, such as differences in allele frequency, genetic architecture, and SNP heritability across ancestries. For instance, rare variants may not be shared across different ancestries, so the actual gains in using larger variant sets from the training population may be undermined when transferring to the other populations.

Cross-population evaluation was carried out by first performing linear scoring on the entire genotype matrix for all samples using plink2 v.2.00a6LM.^17^ With these scores at hand, we then split the samples by the Pan-UKB population labels and evaluated the prediction accuracy within each population separately. The evaluation metric used in all our analyses is incremental $R^{2}$, defined as the difference in the proportion of phenotypic variance explained by a linear model with the polygenic score and covariates and a linear model with covariates only. The covariates used at the evaluation stage include the participant’s age, sex, and the first 20 principal components (PCs). These covariates, in addition to the population labels, are provided in Return 2442 of the UK Biobank.^18^^,^^44^

#### Extracting CARTaGENE genotype and phenotype data and cross-biobank evaluation

To ensure that the trends we observed within the UK Biobank are replicable, we extended our analysis to the CARTaGENE Biobank, a prospective cohort with genotype data on N=29,337 participants from Quebec, Canada.^48^ The majority ancestry among CARTaGENE participants is French-Canadian, although individuals from other ancestries are also represented.

For our analysis, we extracted genotype and phenotype data for all participants in the CARTaGENE cohort. The imputed genotype calls are provided by the biobank in PGEN file format.^17^ We used plink2 v.2.00a6LM^17^ to perform linear scoring and generate polygenic scores for the CARTaGENE participants. The scoring pipeline for CARTaGENE was slightly different from that of the UK Biobank, since the former used genome build hg38 for variant calling, whereas the standard UK Biobank dataset used hg19. To translate the scoring files for the CARTaGENE dataset, we used mapping files published by the UKB-PPP consortium^49^ (see data and code availability).

For the purposes of evaluation, we used PCA coordinates and ancestry clustering results generated in a previous study that examined patterns of genetic variation in CARTaGENE.^50^ In our analysis, we used the top 10 PCs, age, and sex as covariates when computing incremental *R*-squared metrics. To restrict evaluation to European samples, we used UMAP + HDBSCAN clusters corresponding to individuals of European ancestry (N=27,043).^50^

To extract corresponding phenotype data, we manually matched 32 out of the 75 continuous phenotypes that we analyzed in the Pan-UKB. The phenotypes were matched based on their definitions and distributions in both cohorts (Figure S11). Similar to our Pan-UKB pipeline, we excluded outlier phenotypic measurements, primarily those exceeding 3 standard deviations from the mean of each phenotype. Some of the phenotypes were measured on different scales, discretized differently, or showed clear shift in distribution between the CARTaGENE and the UK Biobank. We expect these heterogeneities in phenotypic distribution to affect the portability of UK Biobank-derived PRSs to the CARTaGENE cohort (see discussion).

## Results

### Massive gains in speed and efficiency with streamlined data structures and inference algorithms

To assess the overall impact of the algorithmic improvements introduced in the latest release of the VIPRS software (v.0.1), we systematically compared its performance to a previously published version (v.0.0.4) across four axes of computational and statistical performance: total wallclock time, runtime per iteration, peak memory utilization, and prediction accuracy (see material and methods). In addition to these four metrics, we compared the storage requirements of the proposed CSR LD-matrix format against previously published baselines. The benchmarking experiments were carried out using 5-fold GWAS summary statistics for standing height from the UK Biobank (see data and code availability).^13^ The dataset contains summary statistics for ≈1.1 million HapMap3 variants.^13^

Figure 2A shows dramatic reductions in storage requirements for the CSR LD-matrix format, up to 54-fold improvement in space efficiency when using the int8 data type to represent the entries of the LD matrix. Due to the elimination of redundancy by storing only the upper-triangular portion of the matrix, even the double-precision data type consumes 2.5 times less storage space than the old matrix format (VIPRS v.0.0.4). Combining this triangular format with quantization allowed us to reduce storage requirements by up to an order of magnitude (Figure 2A) relative to published LD-matrix resources from commonly used PRS methods.^10^^,^^11^^,^^12^ Although quantization is a lossy compression technique, the resolution for int8, the most compact data type, is still below 0.01, a value small enough to be generally thresholded by many statistical genetics methods.^9^^,^^51^

Our previous study established that VIPRS v.0.0.4 is one of the fastest model-based PRS methods published to date, achieving an average wallclock time of roughly 15 min on ≈1.1 million HapMap3 variants.^13^ Here we show that, on the same HapMap3 subset, the latest version of the software (v.0.1) is over six times faster in terms of overall wallclock time (Figure 2B) and consumes at least three times less memory (Figure 2C), meanwhile achieving comparable prediction accuracy on standing height in the UK Biobank (Figure 2D). On a dataset of this scale, our benchmarks show that VIPRS v.0.1 converges in less than 60 s and consumes a modest 800 MB of RAM at its peak. Figure 2E largely explains this improved efficiency, as it demonstrates more than an order-of-magnitude improvement in runtime per iteration in the latest version of the software. This scaling does not necessarily translate to total wallclock time because there are other fixed costs beyond the inference step itself, mainly loading the data from disk, harmonizing LD and summary statistics, and loading libraries and other dependencies.

In these experiments, we used both versions of the software with default settings. This means that no multi-threading or parallel processing is in effect and the LD mode for v.0.1 is triangular. To analyze the impact of the other algorithmic changes, we conducted more detailed ablation experiments, which we turn to next.

### Further improvements in scalability with parallelism and compact LD data representation

Scaling PRS methods to tens of millions of variants requires faster coordinate-ascent routines with minimal memory footprint. To understand how the two layers of parallelism that we introduced impact the performance of VIPRS, we examined their impact along two of the axes discussed earlier: runtime per iteration and wallclock time. The benchmarking experiments in this section were done using the same 5-fold GWAS summary statistics for standing height in the UK Biobank (see material and methods and data and code availability).^13^

**Figure 3:**
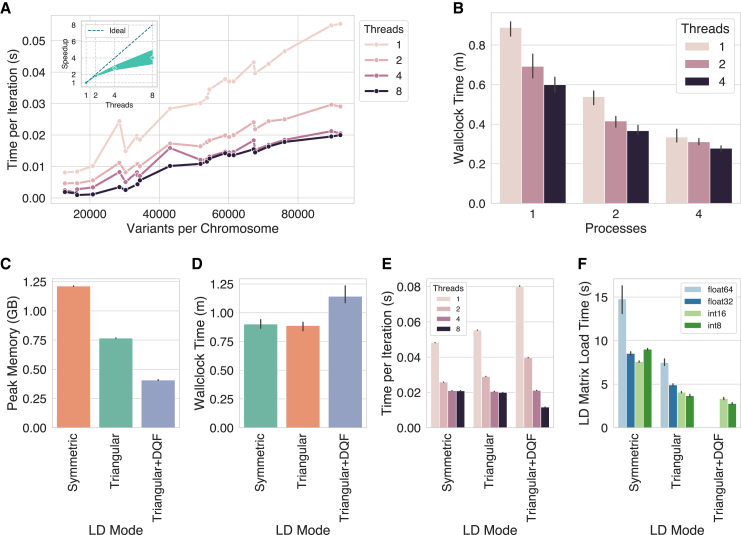
The impact of multi-threading, parallel processing, and LD-matrix representations on the computational performance of VIPRS The benchmarks were run on 5-fold GWAS summary statistics for Standing Height from the UK Biobank with ≈1.1 million HapMap3 variants. (A) Multi-threading across variants. The line plot shows the runtime per iteration (in seconds) as a function of the number of variants on each chromosome (*x* axis) and the number of threads used in the coordinate-ascent updates (colors). The inset shows speedup in runtime per iteration as a function of the number of threads. Dots show average speedup, and bands display smoothed 95% confidence interval across chromosomes. (B) Total wallclock time (minutes) as a function of the number of processes in the process pool (*x* axis) and the number of threads used in the coordinate-ascent step (colors). (C and D) Peak memory (GB) and total wallclock time (minutes) as a function of how the LD matrix is represented in memory. (E) Runtime per iteration (seconds) on chromosome 1 as a function of the LD-matrix representation (*x* axis) and number of threads (colors). (F) Time to load and post-process the LD matrix (seconds) for ≈1.1 million HapMap3 variants from disk to memory, across the three different modes (*x* axis) and storage data types (colors). Vertical black lines above the bars in (B)–(D) and (F) show standard error across the 5-folds.

Figure 3A shows that parallel coordinate ascent significantly improves the runtime per iteration of the algorithm in a way that is commensurate with the number of threads. Scaling is not linear with the number of threads, however, and does vary between chromosomes (inset in Figures 3A and S5). We hypothesize that this is in part due to the fact all threads write to a shared q vector (Figure S1), which can lead to synchronization overhead. Additionally, in some cases, multi-threading performance may be limited by memory bandwidth. We expect bigger benefits of multi-threading for larger chromosomes with relatively small LD blocks, which is consistent with the trends we observe in Figures 3A and S5.

The benefits of multi-threading on total wallclock time are evident, with four threads reducing total runtime by approximately 30% (Figure 3B). This improvement is achieved without affecting prediction accuracy on the held-out test set (Figure S3). As discussed in material and methods, the benefits of multi-threading on wallclock time may be partially dampened in some settings: multi-threaded implementations may require more iterations to reach convergence, especially when the variants/threads ratio is small. As for the second layer of parallelism, multi-processing across chromosomes with process pools shows clear benefits on wallclock time for this dataset, with the reductions in total runtime scaling proportionally with the number of processes (Figure 3B). This is to be expected, since processes have their own resources and copies of the relevant data, and the main overhead here is forking the processes themselves.

The other set of algorithmic improvements introduced here are the memory-efficient implementations of the coordinate-ascent algorithm. Figure 3C shows that the triangular LD mode outlined in algorithm 2 (Figure S1) significantly reduces memory usage compared to the symmetric LD mode (roughly 40% reduction) without affecting total wallclock time, runtime per iteration, or prediction accuracy (Figures 3D, 3E, and S3). Combining triangular LD mode with dequantizing the entries of the LD matrix on-the-fly (DQF) reduces memory utilization by another factor of 2 (Figure 3C), although the overhead of repeatedly dequantizing the data in every iteration results in notable slowdowns in both total wallclock time and runtime per iteration (Figures 3D and 3E). Interestingly, the DQF variant of the algorithm scales better with multi-threading in the coordinate-ascent step (Figure S5), perhaps due to reduced memory bandwidth requirement of the more compact LD data. Finally, Figure 3F shows the time to load the LD matrix depending on the encoded data type and LD mode for the algorithm. The benchmarks demonstrate that dequantizing the data in this case is very swift, although symmetrizing the matrix may approximately double the loading time.

Overall, in the case of a relatively small dataset of ≈1.1 million HapMap3 variants, the differences between these variations of the algorithm may seem small: differences of 30 s are negligible, and 600 MB of RAM is insignificant for modern computing devices. Naturally, though, we expect these relative differences to become consequential when performing inference over tens of millions of variants, as we discuss next.

### Large-scale analyses of Pan-UKB data with up to 18 million variants

To demonstrate the scalability of the proposed algorithmic improvements, we now turn to the Pan-UKB dataset, a comprehensive resource containing GWAS summary statistics for upward of 7,000 phenotypes across six continental ancestry groups.^18^^,^^44^ The QC and association testing methodology is standardized across the entire dataset, making it an attractive resource for systematic PRS analyses. Furthermore, the Pan-UKB marginal association testing pipeline contains data for all well-imputed variants, up to 28 million, allowing us to test the computational performance and potential benefits of Bayesian modeling of the joint effects of more than a dozen million variants. In this section, we primarily focus on training VIPRS on GWAS data from European samples, as this group has the largest sample size in the UK Biobank.

#### Computational efficiency and numerical stability

To assess the scalability of our method, we computed three LD matrices corresponding to three sets of well-imputed variants in European samples (INFO >0.8^18^): 1.4 million HapMap3+ variants, 13.4 million variants (MAF >0.001), and 18 million variants (MAC >20) (Table 3; see material and methods). We also extracted GWAS summary statistics for 75 of the most significantly heritable phenotypes in the UK Biobank, based on QC metrics provided by the Pan-UKB manifest (see Table S1 and data and code availability).

The first result to highlight is the tractability of the proposed compressed LD-matrix format across the three variant sets. Combined with int8 quantization, where entries are stored using a single byte, the proposed format results in highly compact LD matrices for the 1.4 million HapMap3+ variants, occupying less than 400 MB of storage for each of the six ancestry groups defined in the Pan-UKB (Tables 3 and S2). The advantages of this approach are even more apparent for denser variant sets, with the Zarr LD-matrix format requiring only 13.8 GB and 20.7 GB of on-disk storage for matrices with up to 13.5 million and 18 million variants, respectively (Table 3). With int16 quantization, LD matrices become less compressible, requiring up to 118.9 GB of storage for the 18 million variants (Table 3). Nonetheless, this represents a vast improvement over the old storage format, which required >1 TiB of storage for half as many variants (9.8 million).^13^ It is also instructive to compare this proposed format to the SBayesRC approach of decomposing LD matrices into a low-rank representation, which required up to 75 GB of storage for only 7 million variants.^15^ This compressibility should make it easier to store and disseminate large-scale LD matrices at low cost and little overhead.

With these highly compressed LD matrices at hand, we fitted the VIPRS v.0.1 method to the Pan-UKB GWAS summary statistics for the 75 phenotypes described earlier across the three variant sets in European samples. In this analysis, we first examined computational performance as well as the numerical stability and convergence of the inference algorithm. Since LD data storage type (int8 versus int16) had negligible impact on prediction accuracy (Figure S16), we mainly present results from analyses using the int8 data type, which is more computationally tractable (Figure S7). To situate the proposed improvements in the broader context of existing PRS methods, we also compare the performance of the latest version of the VIPRS software against three baseline methods: LDpred2-auto,^12^ VIPRS v.0.0.4,^13^ and SBayesRC.^15^ The latter method is fit using LD reference panels with HapMap3 as well as an expanded variant set with upward of 7 million variants.

**Figure 4:**
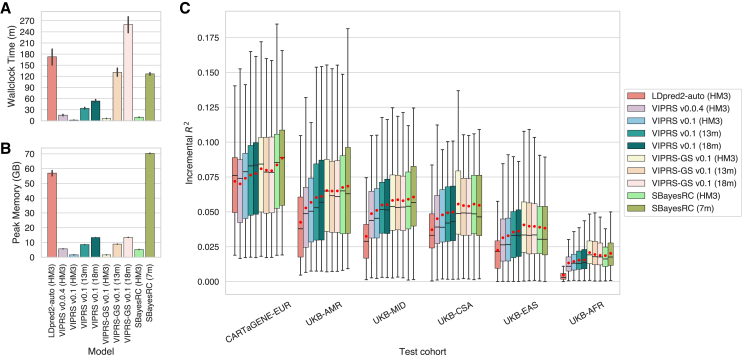
Systematic evaluation of Pan-UKB-trained PRS models across 75 continuous phenotypes and different variant sets All polygenic scores were inferred from European GWAS summary statistics provided by the Pan-UKB initiative. Baseline PRS models included in the analysis are LDpred2-auto, the previous version of VIPRS (v.0.0.4), as well as two versions of the SBayesRC model: SBayesRC (HM3) and SBayesRC (7m), which employ LD reference panels with HapMap3 and 7 million variant sets, respectively. The latest VIPRS models (v.0.1) include two strategies for hyperparameter tuning, Variational EM and grid search (GS), which were trained with three variant sets: HM3 (HapMap3+), 13m (MAF >0.1%), and 18m (MAC >20). (A and B) Average wallclock time (minutes) and peak memory usage (GB) across all 75 phenotypes. Vertical black lines on top of bars show standard errors for each metric. (C) Comparative prediction accuracy (incremental *R*^2^) between the PRS models on six held-out cohorts across two biobanks: UK Biobank (UKB) and CARTaGENE. The ancestry groups are EUR (European), AMR (Admixed American), MID (Middle Eastern), CSA (Central and South Asian), EAS (East Asian), and AFR (African). The box for each method and test cohort shows the quartiles of the $R^{2}$ metric across the 75 phenotypes. Red dots indicate the average of the $R^{2}$ metric for the same phenotypes.

Figure 4A highlights the speed and efficiency of the inference algorithms implemented in VIPRS v.0.1, demonstrating that the method converges in less than an hour and utilizes less than 15 GB of RAM on GWAS summary data for up to 18 million variants. Comparing VIPRS v.0.1 to popular baselines on the HapMap3 subset, we see that it is over 80 times faster than LDpred2 and requires 35 times less memory (Figures 4A and 4B). Compared to SBayesRC, with its low-rank representation for the LD matrix, VIPRS v.0.1 is four times faster and utilizes three times less memory (Figures 4A and 4B). With the larger variant sets, the VIPRS v.0.1 algorithms are significantly more efficient than SBayesRC (7m), utilizing five times less memory and taking half the runtime, despite performing inference over ≈2.4 more variants (Figures 4A and 4B).

Tuning the hyperparameters of the VIPRS model with grid search (VIPRS-GS), using a modestly sized grid of 20 points, increases the runtime of the software by 3- to 4-fold (Figure 4A), with negligible impact on memory usage (Figure 4B). We note that, in these experiments, the runtime of the program is affected by fetching the LD data across the cluster network (Figure S7), a task that should have negligible runtime if the data are stored locally on the compute node. For the 18 million variant set (MAC >20), the task of training/inference requires roughly 40 min genome wide (Figure S7). Additionally, these results are obtained while utilizing modest computational resources for all methods: eight CPU cores. When run on high-performance computing clusters with over 40 cores, the VIPRS algorithms converge in less than 20 min genome wide (Figure S6).

Given the potential for numerical instabilities discussed in material and methods, we examined model convergence when performing inference in ultra-high dimensions. In Figure S12, we show that the incremental *R*-squared metric on the training set improves uniformly across the 75 phenotypes examined. This supports the idea that the algorithm is stable, and the model converges well in these high-dimensional settings, although it does not necessarily imply that these larger models will extrapolate better.

#### Accuracy and transferability of highly parameterized PRS models

To test the extrapolation ability of the inferred PRS models, we assessed the prediction accuracy on six held-out test cohorts across two biobanks (see material and methods). In the first case, we examined within-ancestry but cross-biobank transferability, where UK Biobank-derived PRS models trained on European GWAS data were evaluated on European samples in the CARTaGENE biobank.^48^ In the second case, we examined the transferability of these same PRS models on five held-out ancestry groups in the Pan-UKB (Table 2).^18^

Figure 4C illustrates the prediction accuracy of the four PRS methods and their variations on the 75 phenotypes and across the six test cohorts. In the cross-biobank case (CARTaGENE-EUR), it appears that all methods have comparable prediction accuracy, with the fully Bayesian SBayesRC model slightly outperforming other methods. Using denser variant sets marginally improves the predictive performance of both SBayesRC and VIPRS v.0.1, although this benefit does not extend to the grid search version of VIPRS (see discussion). The impact of using denser variant sets on within-ancestry prediction accuracy appears to be rather small (3%–5% on average). This is consistent with the magnitude of the improvement reported in recent analyses, at least for methods that do not utilize functional annotations^15^ (see discussion).

In the cross-ancestry case, we observed more pronounced differentiation in the predictive performance of the models, with the LDpred2-auto model significantly lagging behind other methods for most ancestry groups in the UK Biobank (Figure 4C). Aside from the poor cross-ancestry transferability of LDpred2-auto, other methods achieved comparable prediction accuracy on the held-out ancestries, with the top-performing methods being VIPRS-GS and SBayesRC. Notably, these two top-performing models do not appear to significantly benefit from using denser variant sets on average (Figure 4C; see discussion). On the other hand, using denser variant sets improves the performance of the standard EM version of VIPRS v.0.1 by 6% on average and upward of 9.5% in the case of East Asian samples (UKB-EAS). However, this improvement is not sufficient to bridge the gap with the top-performing models in most cases (see discussion).

Overall, our analyses demonstrated that VIPRS v.0.1 is capable of performing inference over millions of genetic variants and producing highly accurate and robust polygenic scores. This competitive accuracy is achieved while utilizing minimal computational resources compared to baseline methods.

## Discussion

In this work, we presented an integrated suite of data structures, algorithms, and software tools to scale model-based PRS inference to millions of genetic variants, meeting the ever-increasing depth and resolution of modern GWASs.^52^ Our technical contributions extend from the practical solutions for large-scale LD-matrix computation and storage to robust and efficient coordinate-wise optimization algorithms that can perform variational Bayesian regression^13^ over a dozen million variants in as little as 20 min and using less than 15 GB of RAM.

LD matrices are an integral part of modern statistical genetics toolkits, playing an essential role in fine-mapping,^23^^,^^53^ PRS inference,^8^^,^^10^^,^^11^^,^^12^^,^^13^^,^^15^^,^^25^ and imputation or simulation of summary statistics,^19^^,^^54^ among other applications. Previous work has explored various techniques for computing, sparsifying, and transforming LD matrices,^8^^,^^11^^,^^13^^,^^16^^,^^22^^,^^25^^,^^55^ with the aims of making downstream inference algorithms more efficient, numerically stable, and accurate. Despite this, clear and universal standards for representing LD matrices are lacking, leading each method to develop its own set of tools and storage formats. In this work, we attempted to pool together best practices from disparate subfields of statistical genetics to present a universal, cloud-native storage format, with compression ratios of up to 50-fold over naive approaches. This compactness rivals recent low-rank representations of LD matrices^15^ while being a more universal representation that can readily serve a wide variety of applications. It is also competitive with a recently proposed, ultra-sparse estimator of precision matrices (inverse of LD),^19^ making it useful for many applications that require access to pairwise correlations between genetic variants. Compressibility of LD matrices provides many practical benefits besides allowing for more efficient inference algorithms. For one, it reduces the barrier for computing and releasing LD matrices for fine-grained subgroups in large cohorts or biobanks, potentially improving the quality of PRS inference in some minority populations.^16^ Recent efforts to release comprehensive LD matrices as a public resource proved to be costly, requiring terabytes of storage.^18^^,^^56^ While cloud storage costs are relatively minor, moving data of this scale along global or local networks can still incur substantial costs.

Furthermore, our analysis highlighted the importance of the spectral properties of LD matrices on the accuracy and numerical stability of PRS inference algorithms.^39^^,^^40^ Concretely, our analyses outlined how the spectral properties of an LD matrix are affected by many common choices for estimating pairwise correlations between genetic variants. These choices include the definition of sparsification mask (e.g., banded or block-diagonal), estimation in the presence of missing genotype calls, inclusion of variants in long-range LD regions, thresholding, and approximation errors. While we provided practical recommendations for computing well-conditioned matrices that behave stably even in ultra-high dimensions, there remain many open questions with regard to the precise balance between compression, shrinkage, and accuracy that we leave for future work.

The compressed LD-matrix format integrates seamlessly with the updated coordinate-ascent inference algorithms, allowing us to derive efficient coordinate-wise update rules that operate on compressed and non-redundant LD data. This “low-memory” version of the VIPRS method reduces memory usage during inference by more than an order of magnitude compared to a naive implementation while achieving comparable prediction accuracy. Our work further demonstrates that these update rules can be easily parallelized without assuming any special structure, such as independent LD blocks, leading to significant improvements in inference speed. Notably, we found that combining parallel coordinate ascent with compact LD representations, i.e., quantization, not only saved memory but also enhanced scaling performance (Figures 3 and S5).

This tangible boost in processing speed and memory efficiency enabled testing the limits of model-based PRS inference in a systematic manner, using GWAS summary statistics for up to 18 million well-imputed variants across 75 of the most significantly heritable phenotypes in the Pan-UKB.^18^^,^^44^ Comparing the latest version of VIPRS to popular baseline methods revealed that it is 5–80 times more efficient while attaining prediction accuracy on par with the best-performing methods. Similar to our previous work,^13^ we demonstrated that tuning some of the hyperparameters of VIPRS via grid search significantly improved its predictive performance. While grid search increases the runtime of the method by a factor of 3–4, it remains considerably faster and more memory efficient than competing methods.

Our results indicated that using dense variant sets can marginally but consistently enhance the accuracy of some PRS models but not others. For instance, performing inference over 18 million variants with the standard VIPRS v.0.1 method improved its accuracy on the scale of 10%–15% in some ancestry groups relative to the HapMap3+ subset. However, this gain in predictive performance does not fully bridge the gap with more capable models, such VIPRS-GS and SBayesRC. Meanwhile, these capable models, especially VIPRS-GS, do not seem to benefit from using dense variant sets in our cross-ancestry analyses. We hypothesize that this may be partly due to overfitting (Figure S13),^57^ poor approximation of validation summary statistics in high dimensions,^31^ or other numerical errors that may become more prominent in large-scale regression.

The cross-biobank and within-ancestry analysis on the CARTaGENE-EUR cohort also showed marginal benefit to using dense variant sets in some cases, although most models perform similarly in this setting. This suggests that, in European samples at least, HapMap3 markers tag most causal variants for these phenotypes rather well. Other factors could have dampened the impact of using more dense variant sets in this particular case. For instance, some of the manually matched phenotypes showed significant differences in their distribution across the two biobanks (Figure S11), introducing potential batch effects. Furthermore, while both biobanks used similar genotyping arrays for their respective cohorts,^44^^,^^48^ imputation pipelines and reference panels are different,^44^^,^^48^ which might lead to systematic biases in the imputation of some causal variants.

From a broader perspective, these results suggest that factors such as model assumptions or priors, inference techniques, and quality of input data potentially have greater impact on PRS accuracy than the scale or density of the LD reference panel itself. This observation is consistent with the conclusions from the SBayesRC paper^15^: primarily, benefits of dense variant sets are more pronounced when combined with priors informed by genomic annotations. Thus, a natural direction from this work is to extend the VIPRS model to incorporate more flexible and functionally informed priors.

Finally, many of the gains in efficiency and scalability presented here are not restricted to the VIPRS inference algorithms per se and should be widely applicable. Many coordinate-wise optimization or sampling-based methods, such as Lassosum^8^ and LDPred,^12^^,^^25^ can benefit from the “low-memory” updating scheme in addition to unrestricted parallelism to improve computational efficiency, as demonstrated in a recent paper from our group.^58^ Although parallel coordinate descent algorithms for the LASSO have been extensively studied,^30^ there is also comparable, though less well known, literature on parallel Gibbs sampling algorithms.^59^ The cloud-native Zarr format, which powers our LD-matrix storage specification, comes with public APIs in many popular programming languages, which should make it relatively easy to access and build on the LD storage format presented in this work.

In summary, our updated VIPRS software offers orders-of-magnitude improvements in storage, computation, and memory efficiency, requiring only 20 min to infer the joint effects of all well-imputed bi-allelic variants in the UK Biobank. These improvements situate it as the next-generation PRS tool in the modern pipeline of increasingly large-scale and comprehensively phenotyped and genotyped biobanks.

## Data and code availability

With the exception of individual-level biobank data from the UK and CARTaGENE biobanks, all the data and software used in this study are publicly available via the following URLs.

• Scripts to replicate all the analyses and generate the figures for this paper are available via GitHub: https://github.com/shz9/viprs-benchmarks-paper
• The magenpy python package for computing LD matrices/harmonizing LD and GWAS summary statistics: https://github.com/shz9/magenpy
• Latest version of the VIPRS software: https://github.com/shz9/viprs
• The Zarr library used to store compressed LD matrices: https://zarr.readthedocs.io/en/stable/
• A Google Colab notebook illustrating the speed and main features of the CLI of the viprs software: https://github.com/shz9/viprs/blob/master/notebooks/viprs_cli_example.ipynb
• Script to convert between old and proposed CSR format for the VIPRS LD matrices: https://github.com/shz9/magenpy/blob/master/examples/convert_old_ld_matrices.py
• SBayesRC software and LD reference panels: https://github.com/zhilizheng/SBayesRC
• LDpred2 software and LD reference panels: https://privefl.github.io/bigsnpr/articles/LDpred2.html
• LD matrices for the six continental ancestry groups are available for download via GitHub (https://github.com/shz9/viprs/releases/tag/v0.1.2) and Zenodo (https://zenodo.org/records/14614207).
• Five-fold benchmarking summary statistics for standing height: https://doi.org/10.5281/zenodo.14270953
• LD blocks defined by LDetect: https://bitbucket.org/nygcresearch/ldetect-data/src/master/
• LD matrices for older versions of VIPRS (v.0.0.4): https://doi.org/10.5281/zenodo.7036625
• The Pan-UKB phenotype manifest with heritability estimates, QC flags, and hyperlinks to download GWAS summary statistics: https://docs.google.com/spreadsheets/d/1AeeADtT0U1AukliiNyiVzVRdLYPkTbruQSk38DeutU8
• Mapping files for rsids across genome builds hg19 and hg38 from UKB-PPP: https://www.synapse.org/Synapse:syn51364943/wiki/622119.

## Acknowledgments

We thank members of the Li and Gravel labs for useful feedback and discussions on earlier drafts of the manuscript. This research was supported by the following funding programs: the Canada Research Chair (Tier 2) in Machine Learning for Genomics and Healthcare (CRC-2021-00547) and 10.13039/501100000038Natural Sciences and Engineering Research Council of Canada (NSERC) discovery grant (RGPIN-2016-05174) to Y.L.; the 10.13039/501100000024Canadian Institutes of Health Research (CIHR) project grant 437576, 10.13039/501100000038NSERC grant RGPIN-2017-04816, and the Canada Research Chair program to S.G.; and the Canada Foundation for Innovation. This research used the NeuroHub infrastructure and was undertaken thanks in part to funding from the 10.13039/501100010785Canada First Research Excellence Fund, awarded through the Healthy Brains, Healthy Lives Initiative at McGill University. This research was enabled in part by support provided by Calcul Québec and the 10.13039/501100021202Digital Research Alliance of Canada.

## Author contributions

Y.L., S.G., and S.M. supervised the work. Under the supervision of Y.L. and S.M., S.Z. and C.A.H. benchmarked the software, analyzed computational bottlenecks, and tested various solutions to speed up the coordinate-ascent step. S.Z. implemented the proposed LD storage format and associated data structures and software utilities. S.G. and S.Z. examined the spectral properties of LD matrices and their impact on model convergence and stability. C.A.H. and S.Z. conducted the benchmarking experiments. S.Z. conducted the Pan-UKB analyses. S.Z. wrote the initial manuscript. All authors edited and reviewed the final version.

## Declaration of interests

The authors declare no competing interests.

## Web resources

CARTaGENE Biobank Application 936028, https://cartagene.qc.ca/en/
UK Biobank Application 45551, https://www.ukbiobank.ac.uk/

## Supplemental information

Document S1. Figures S1–S17 and Tables S1 and S2

Document S2. Article plus supplemental information
