# Supplementary material for: Toward whole-genome inference of polygenic scores with fast and memory-efficient algorithms
Source: Am J Hum Genet. 2025 May 26;112(7):1528–46. doi: 10.1016/j.ajhg.2025.05.002 (PMC12256920; doi:10.1016/j.ajhg.2025.05.002)
Supplement: Document S1. Figures S1–S17 and Tables S1 and S2 [file mmc1.pdf]

The American Journal of Human Genetics, Volume *112*

Supplemental information

Toward whole-genome inference of polygenic scores with fast and memory-efficient algorithms

Shadi Zabad, Chirayu Anant Haryan, Simon Gravel, Sanchit Misra, and Yue Li

S1 Supplementary Figures

Coordinate Ascent Variational Inference Algorithms for GWAS summary statistics

Common Inputs

Global Parameters:

t: Current iteration

N: Number of samples

M: Number of genetic variants

σ_ϵ^2: Residual variance

τ_β: Prior precision (inverse variance)

π: Prior probability of non-zero effect size

Variant-specific Parameters:

$\hat{\beta}_j$: Marginal GWAS effect size

$\eta_j^{(t-1)}$: Posterior mean effect size

$q_j^{(t-1)}$: q-factor ($q_j := \sum_{i \neq j} R_{ij}\eta_i$)

Algorithm 1: Symmetric LD

Specific Input:

R: Symmetric LD matrix

for *j = 1 to M* **do**

```
# Update posterior precision
```
$\tau_j^{(t)} \leftarrow \frac{N+\sigma_\epsilon^2\tau_\beta}{\sigma_\epsilon^2}$
```
# Update posterior Gaussian mean
```
$\mu_j^{(t)} \leftarrow \frac{N}{\tau_j^{(t)}\sigma_\epsilon^2} \cdot (\hat{\beta}_j - q_j)$
```
# Update posterior inclusion
probability (PIP)
```
$\gamma_j^{(t)} \leftarrow Sigmoid\Big(\log\left(\frac{\pi}{1-\pi}\right) + \frac{1}{2}\log\left(\frac{\tau_\beta}{\tau_j^{(t)}}\right) + \frac{1}{2}\tau_j^{(t)} \cdot (\mu_j^{(t)})^2\Big)$
```
# Calculate change in expected
effect size
```
$\eta_j^{(diff)} \leftarrow \gamma_j^{(t)} \cdot \mu_j^{(t)} - \eta_j^{(t-1)}$
```
# Update q-factor for all
neighboring variants
```
for $i \neq j$ **do**

$q_i \leftarrow q_i + R_{ji} \cdot \eta_j^{(diff)}$

end
```
# Update expected effect for
next iteration
```
$\eta_j^{(t)} \leftarrow \eta_j^{(t-1)} + \eta_j^{(diff)}$

end

Algorithm 2: Triangular LD

Specific Input:

$R^{(UT)}$: Upper-triangular LD matrix

for *j = 1 to M* **do**

```
# Update posterior precision
```
$\tau_j^{(t)} \leftarrow \frac{N+\sigma_\epsilon^2\tau_\beta}{\sigma_\epsilon^2}$
```
# Update posterior Gaussian mean
```
$\mu_j^{(t)} \leftarrow \frac{N}{\tau_j^{(t)}\sigma_\epsilon^2} \cdot (\hat{\beta}_j - q_j)$
```
# Update posterior inclusion
probability (PIP)
```
$\gamma_j^{(t)} \leftarrow Sigmoid\Big(\log\left(\frac{\pi}{1-\pi}\right) + \frac{1}{2}\log\left(\frac{\tau_\beta}{\tau_j^{(t)}}\right) + \frac{1}{2}\tau_j^{(t)} \cdot (\mu_j^{(t)})^2\Big)$
```
# Calculate change in expected
effect size
```
$\eta_j^{(diff)} \leftarrow \gamma_j^{(t)} \cdot \mu_j^{(t)} - \eta_j^{(t-1)}$
```
# Update q-factor for subsequent
neighboring variants only
```
for $i > j$ **do**

$q_i \leftarrow q_i + R_{ji}^{(UT)} \cdot \eta_j^{(diff)}$

end
```
# Update expected effect for
next iteration
```
$\eta_j^{(t)} \leftarrow \eta_j^{(t-1)} + \eta_j^{(diff)}$

end
```
# Update q-factor again to reflect
changes to preceding variants
```
$\boldsymbol{q} \leftarrow \boldsymbol{q} + \boldsymbol{R}^{(UT)}\boldsymbol{\eta}^{(diff)}$

Figure S1: Coordinate ascent Variational Inference algorithms for PRS inference from GWAS summary statistics. Symbols highlighted in red denote main differences between the triangular and symmetric LD algorithms.

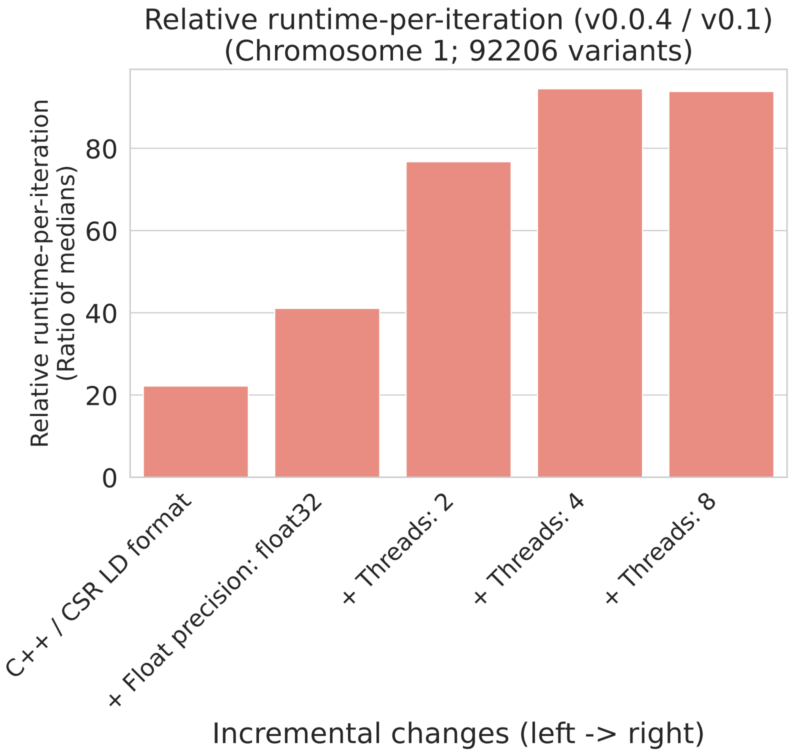

Figure S2: Relative runtime-per-iteration (E-Step runtime) with HapMap3 variants on chromosome 1 for versions 0.0.4 and v0.1 of the `VIPRS` software. The x-axis shows incremental updates to the software from left to right, starting from the updated layout for the LD matrix / implementation of the E-Step in C++, proceeding to using single precision floats (`float32`) for the model parameters, and ends with gains due to the parallel coordinate ascent, with 2, 4, and 8 threads. The y-axis shows the ratio of median runtime-per-iteration for the two versions of the software, where the median is taken across 15 independent runs for each version: median(runtime_v0.0.4) / median(runtime_v0.1). The higher the ratio, the greater the speed gains of the latest implementation over the old.

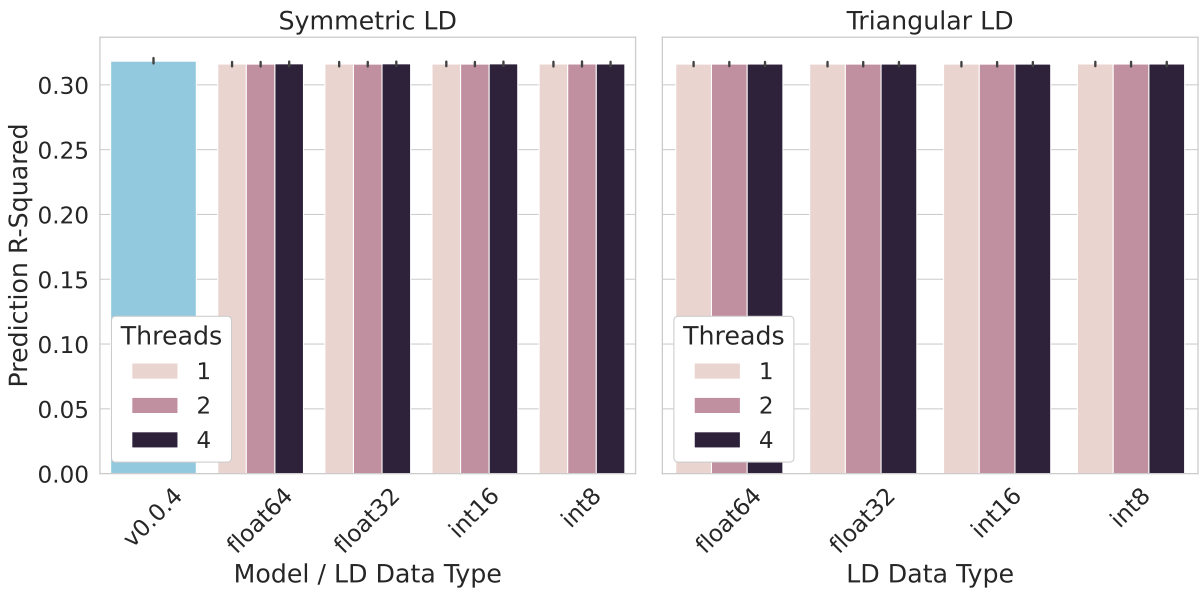

Figure S3: Prediction accuracy for Standing Height in the UK Biobank as a function of LD Mode / LD Data Type / and the number of threads used in the Coordinate Ascent step of `VIPRS` v0.1. The left panel shows Prediction R-Squared for the symmetric LD version of the algorithm and the right panels shows the same metric for the triangular LD version of the algorithm. X-axis shows the LD data types (`float64`, `float32`, `int16`, `int8`) as well as the v0.0.4 version of the software on the far left of the left panel. Threads used in the multi-threaded implementation of `VIPRS` v0.1 are shown in color. Black lines above the bars show standard error across the 5 folds.

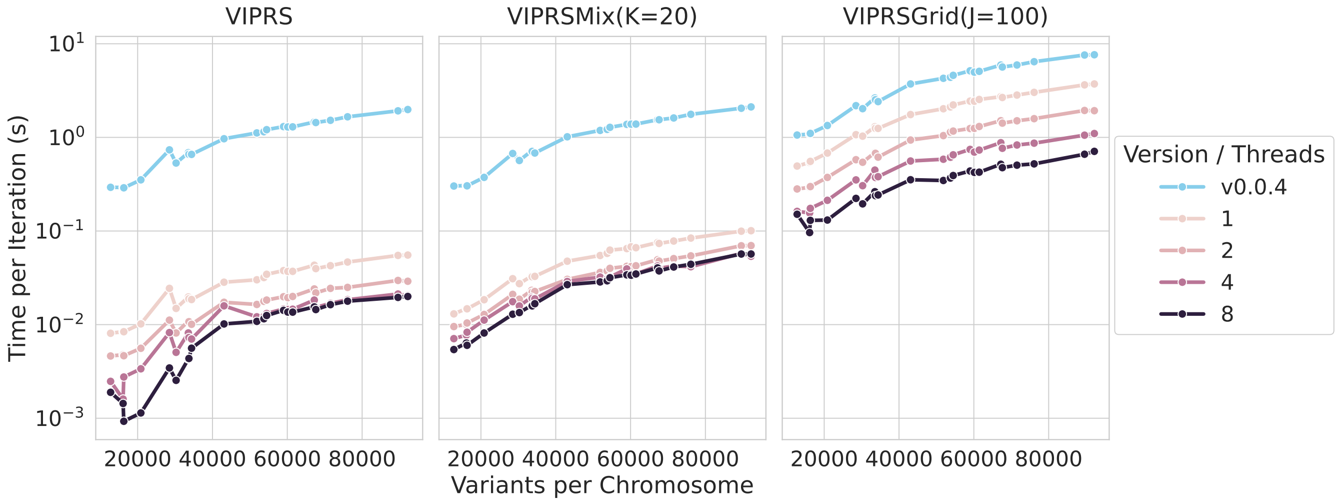

Figure S4: Mean runtime-per-iteration (in second and on log-scale) as a function of the number of SNPs for all the three configurations of the `VIPRS` model. The lines show the performance across versions v0.0.4 (skyblue) and v0.1 with different number of threads (color gradient). Each panel shows a different setup/configuration of the `VIPRS` model. Leftmost panel shows the runtime-per-iteration of the vanilla `VIPRS` with the spike-and-slab prior. Second panel in the middle shows the runtime-per-iteration of `VIPRS(K=20)`, which uses the sparse mixture prior with 20 components. Rightmost panel shows the runtime-per-iteration of the `VIPRSGrid(J=100)` model, which performs inference over 100 different hyperparameter settings.

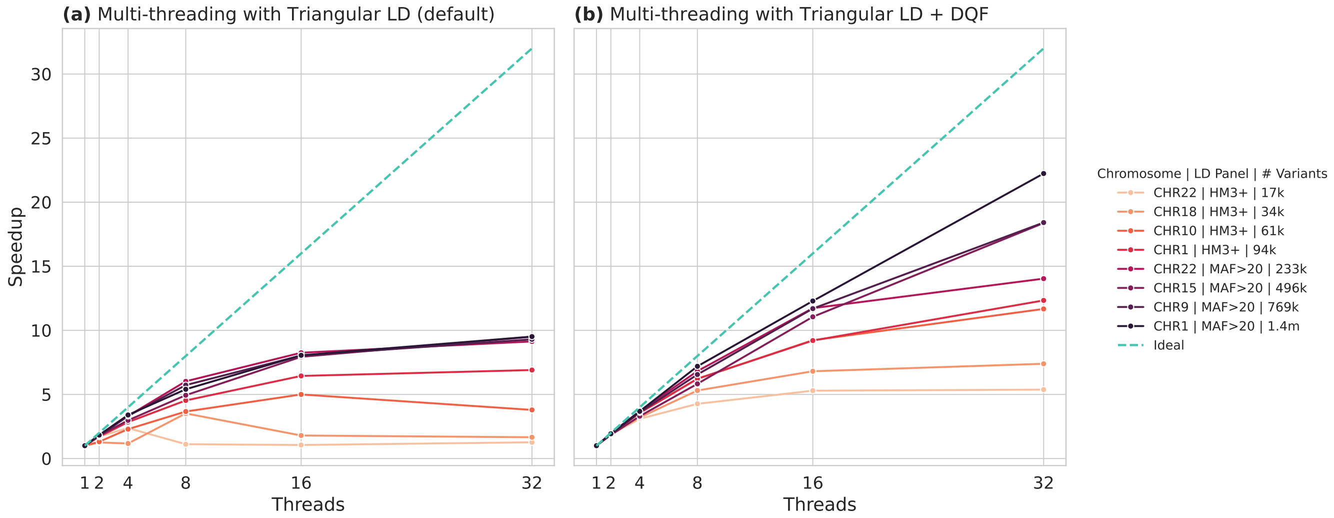

Figure S5: Speedup in runtime-per-iteration as a function of the number of threads across chromosomes of different sizes and two variants sets. Panel **(a)** shows speed up when using default LD mode (Triangular LD) and Panel **(b)** shows scaling when using Triangular LD + dequantize-on-the-fly (DQF) option. Each bold line corresponds to a combination of a chromosome (e.g. CHR1 is Chromosome 1) and variant set: HM3+ (HapMap3+) and MAC>20 (18m). Darker colors denote larger and denser chromosomes, where we expect to see more benefit from multi-threading. Dashed-line shows the ideal speedup expected if the runtime-per-iteration scales linearly with the number of threads.

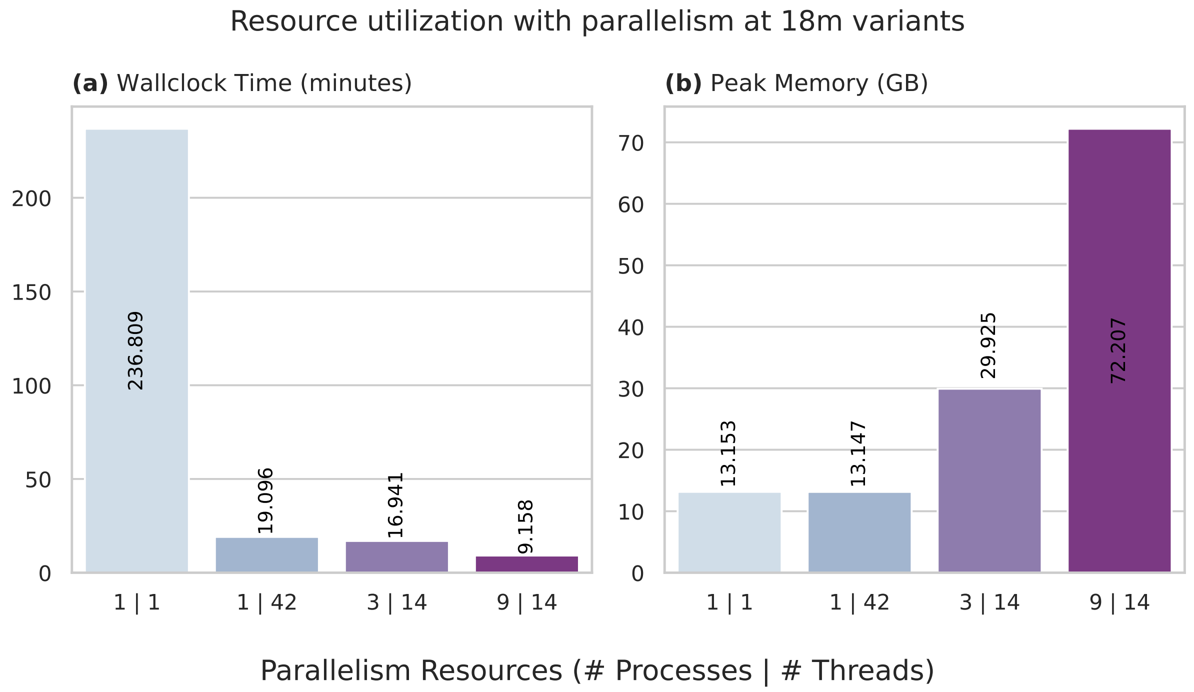

Figure S6: Resource utilization as a function of the number of cores and parallelism strategy (processes vs. threads) when performing inference using the MAC>20 (18 million) variant set. The experiments were conducted using GWAS summary statistics for Standing Height. Panel **(a)** shows total wallclock time as a function of resource configuration (number of processes vs. number of threads). Panel **(b)** shows peak memory (GB) as a function of the same configurations. Multi-threading improves total wallclock time without significantly affecting memory utilization.

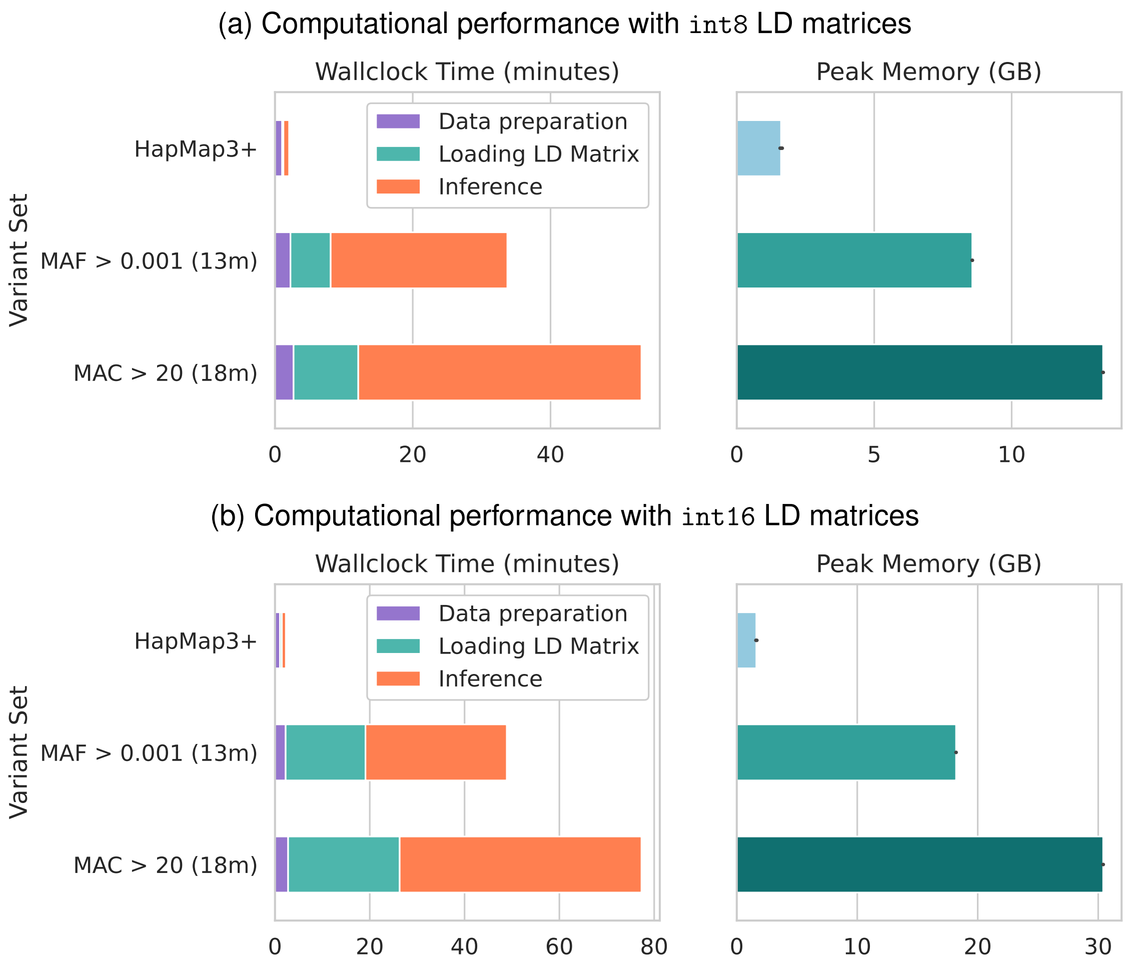

Figure S7: Computational characteristics of performing PRS inference using `VIPRS v0.1` on 75 continuous phenotypes in the Pan-UKB and across three variant sets. Here we use LD matrices stored using both **(a)** `int8` and **(b)** `int16` quantization. Left panels show the average wallclock time (minutes) and right panels show peak memory usage (GB) across all the phenotypes. Colors in panel **(a)** denote average time required for each sub-task during inference. Total wallclock time is mainly slowed due to increased time to fetch and filter the LD data. As expected, memory utilization with `int16` is roughly twice what we obtained with `int8` quantization.

(a) Minimum eigenvalue of windowed LD matrices for European samples ($N = 362446$) in the UK Biobank

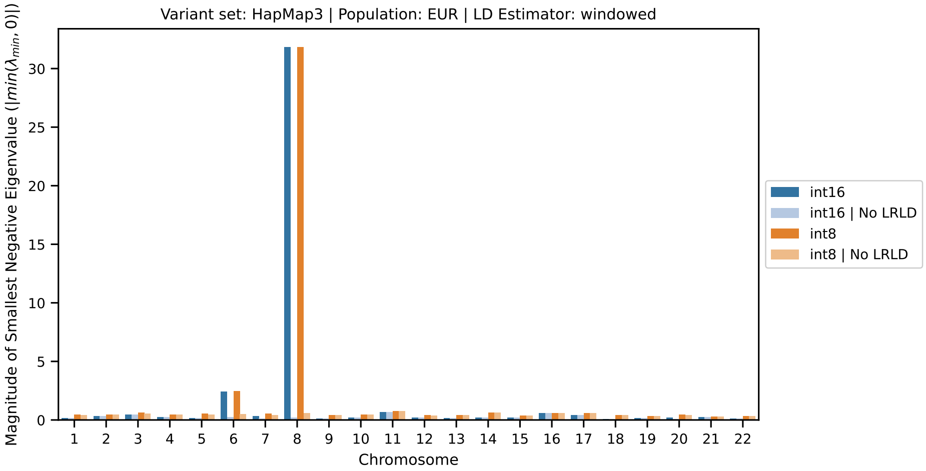

(b) Minimum eigenvalue of windowed LD matrices for East Asian samples ($N = 2700$) in the UK Biobank

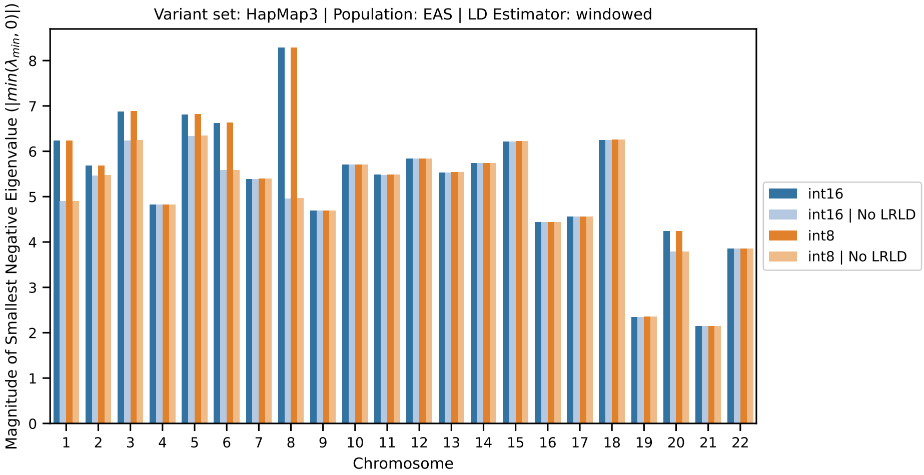

(c) Minimum eigenvalue of windowed LD matrices for African samples ($N = 6255$) in the UK Biobank

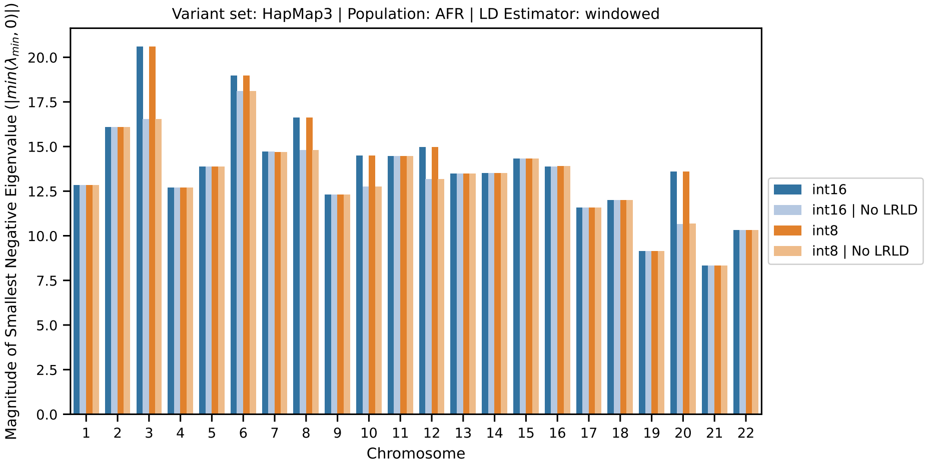

Figure S8: Absolute value of minimum (negative) eigenvalue for windowed (i.e. banded) LD matrices computed for three populations in the UK Biobank. The LD matrices were computed with window size of 3 centi Morgan. Colors denote data type used to store LD matrices: `int16` (blue) and `int8` (orange). Color shades denote LD matrices with (dark) and without (light) variants in long-range LD regions.

(a) Minimum eigenvalues for block-diagonal matrices using HapMap3+ variant set

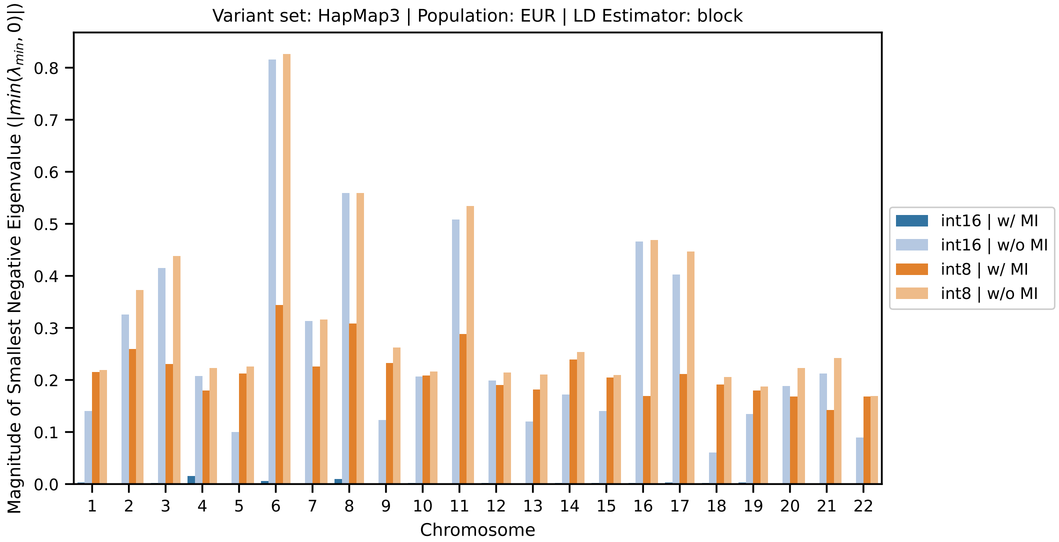

(b) Minimum eigenvalues for block-diagonal matrices using MAF > 0.001 (13m) variant set

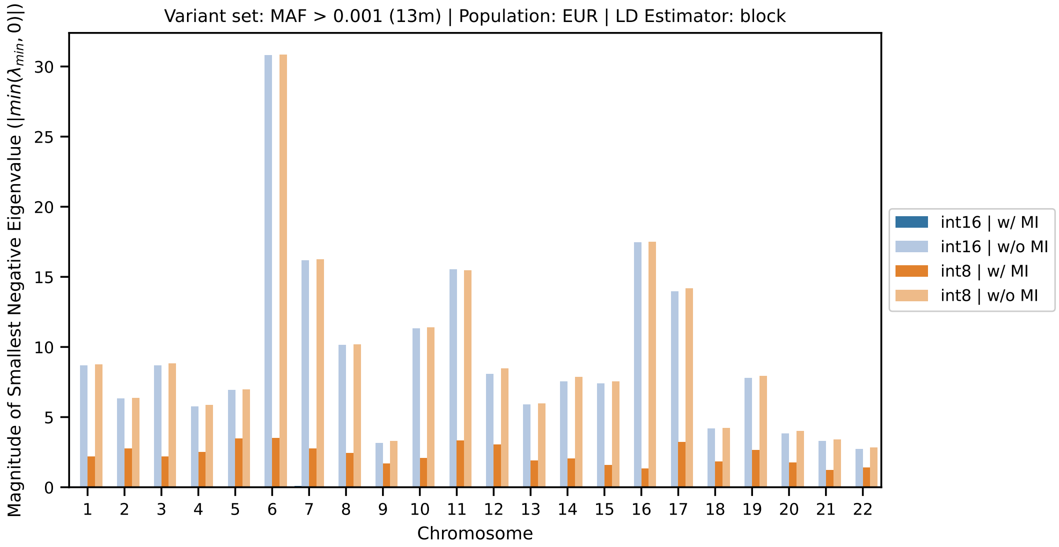

Figure S9: Absolute value of minimum (negative) eigenvalue for block-diagonal LD matrices computed for European samples in the UK Biobank across two variant sets: HapMap3+ (1.4m) and MAF>0.001 (13m). Colors denote data type used to store LD matrices: `int16` (blue) and `int8` (orange). Color shades denote whether Mean Imputation (MI; dark) was used to impute missing genotypes or missing observations were discarded (light).

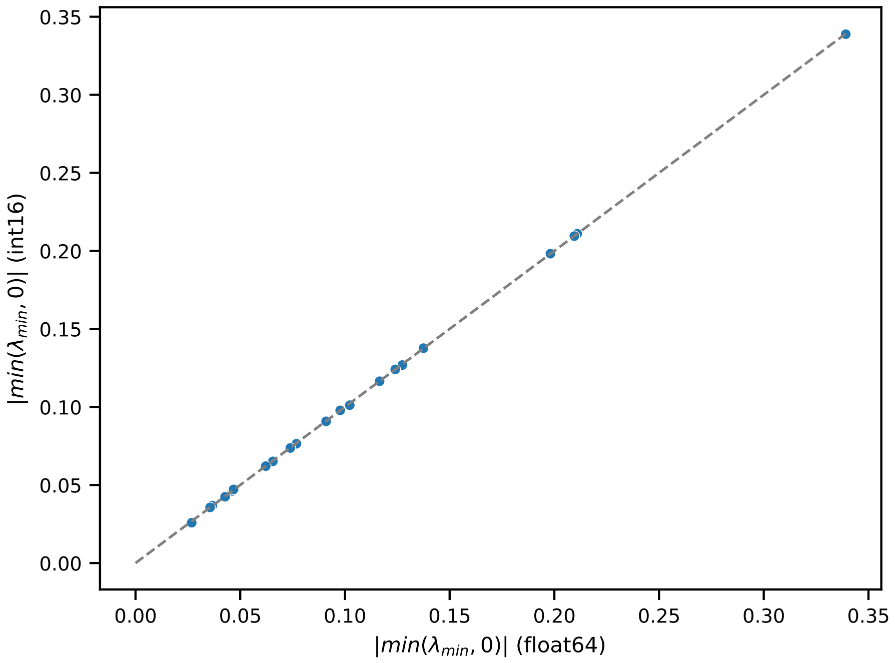

Figure S10: Absolute value of minimum (negative) eigenvalue for windowed HapMap3+ LD matrices computed for European samples in the UK Biobank and stored in two data types: `float64` (x-axis) and `int16` (y-axis). Each dot represents a different chromosome. This figure illustrates that the quantized `int16` data type has negligible impact on the spectrum of LD matrices compared to `float64`.

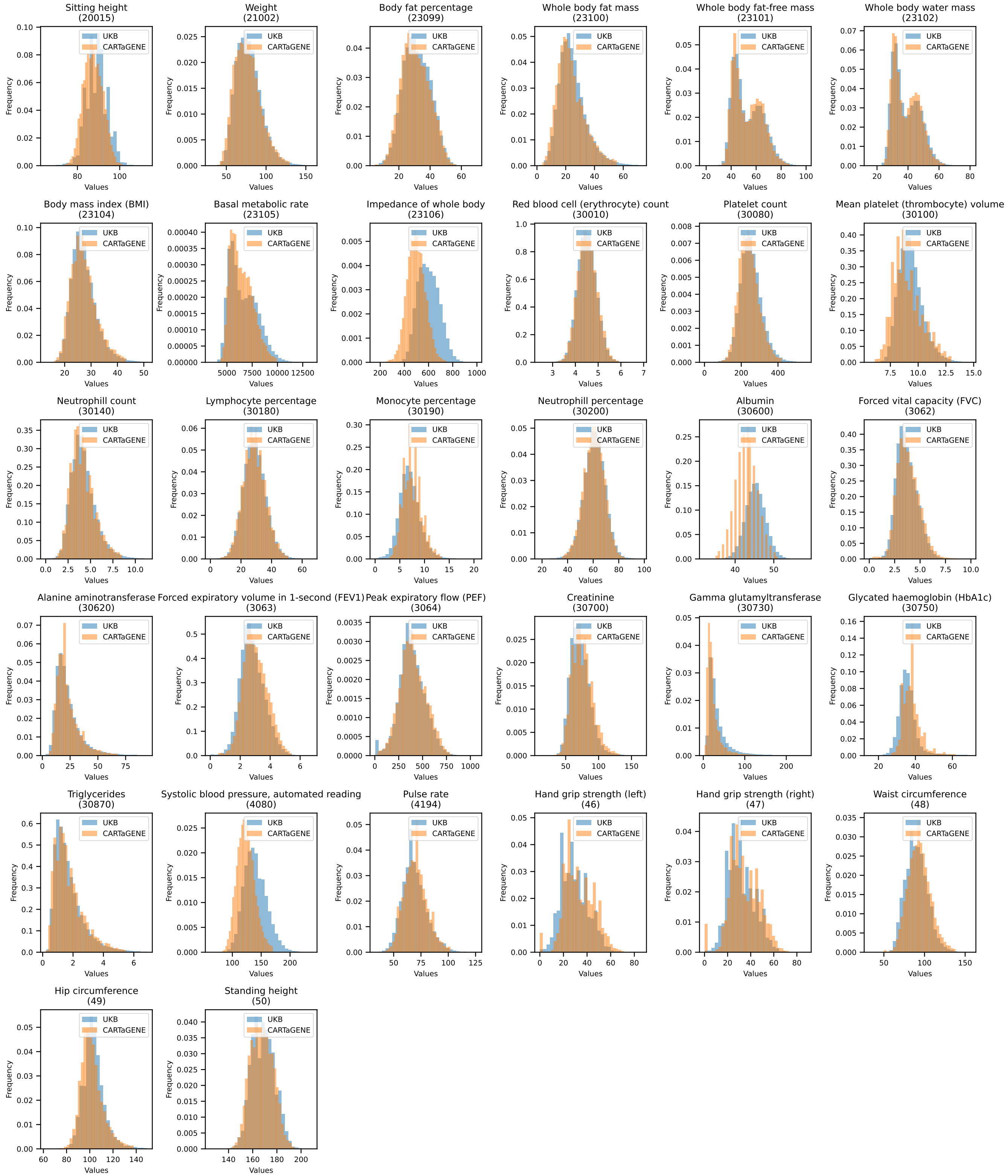

Figure S11: Cross-biobank phenotype distribution comparison between samples in the UK Biobank and the CARTaGENE cohort (Quebec, Canada) across the 32 of the 75 most heritable phenotypes in the Pan-UKB resource. Each panel shows the distribution for a particular phenotype and colored histograms show distribution in UKB (blue) and CARTaGENE (orange) samples.

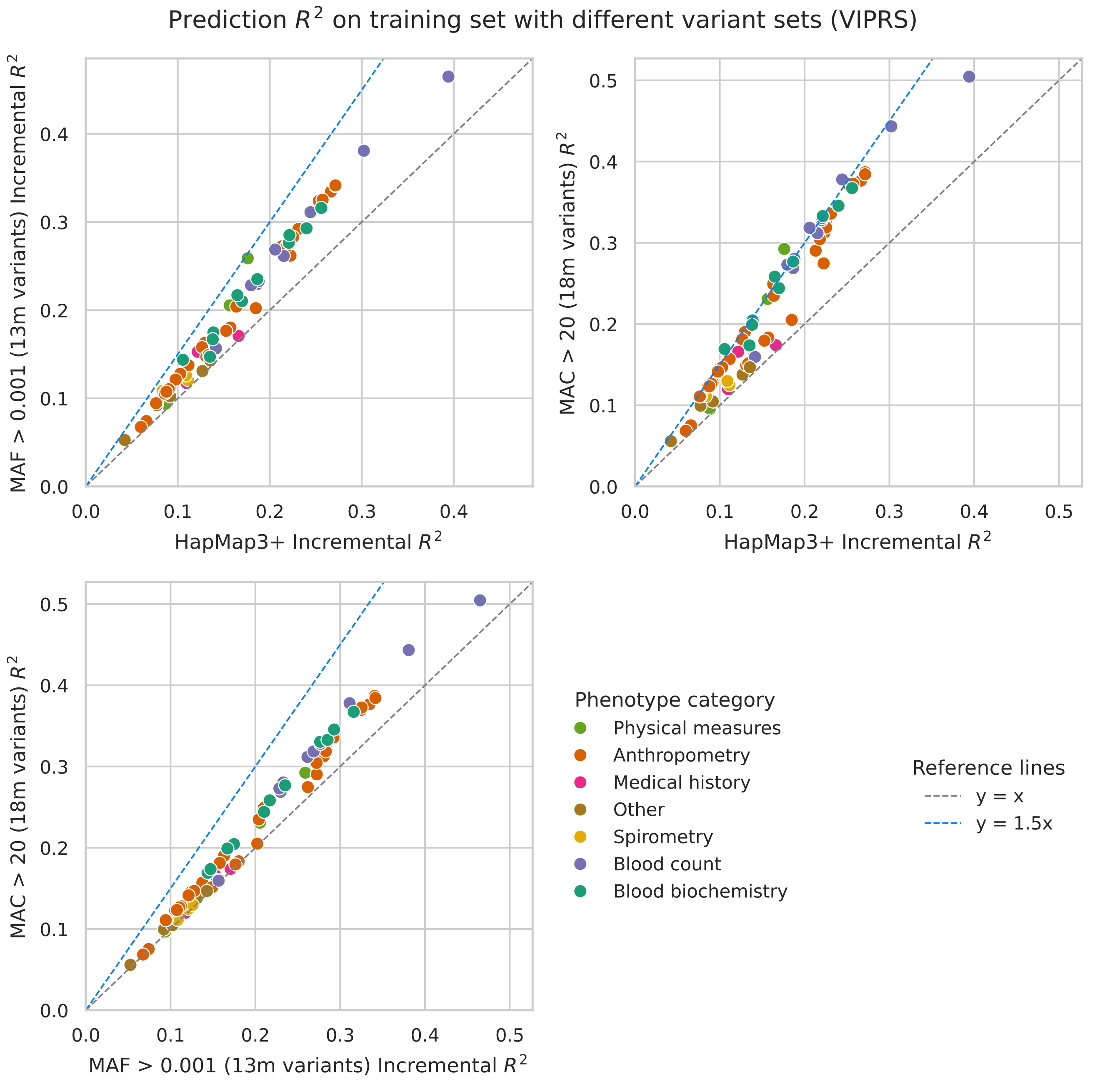

Figure S12: Comparison of prediction accuracy of the `VIPRS v0.1` model on the training cohort (EUR) in the Pan-UKB data resource using three variant sets: HapMap3+, MAF > 0.001 (13m), and MAC > 20 (18m). In each panel, we compare prediction accuracy (incremental R^2) on European samples in the UK Biobank when training PRS models using two of the three variant sets. Each dot shows prediction accuracy for one of the 75 phenotypes and colors denote the phenotype category.

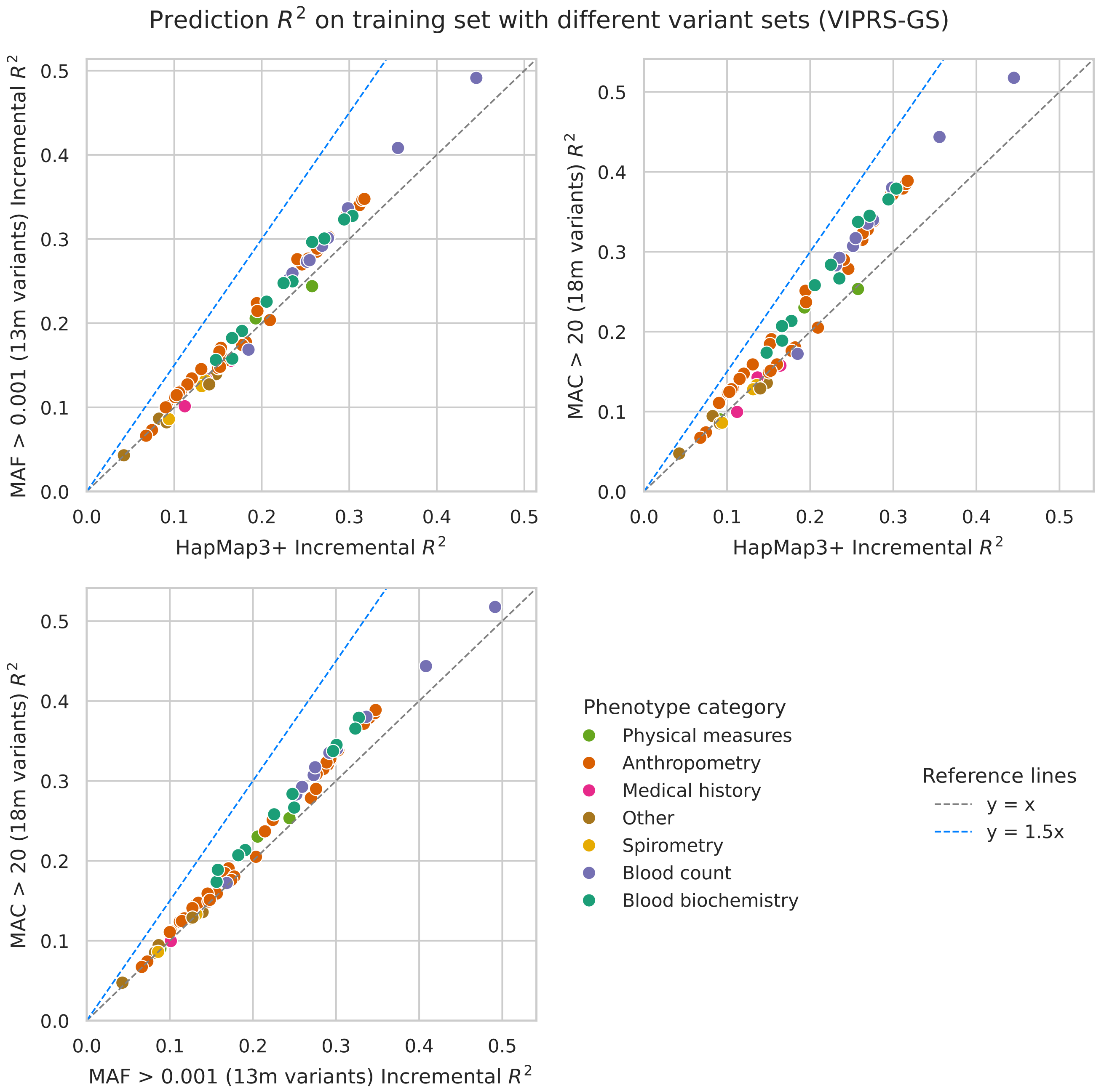

Figure S13: Comparison of prediction accuracy of the `VIPRS-GS v0.1` model on the training cohort (EUR) in the Pan-UKB data resource using three variant sets: HapMap3+, MAF > 0.001 (13m), and MAC > 20 (18m). In each panel, we compare prediction accuracy (incremental R^2) on European samples in the UK Biobank when training PRS models using two of the three variant sets. Each dot shows prediction accuracy for one of the 75 phenotypes and colors denote the phenotype category.

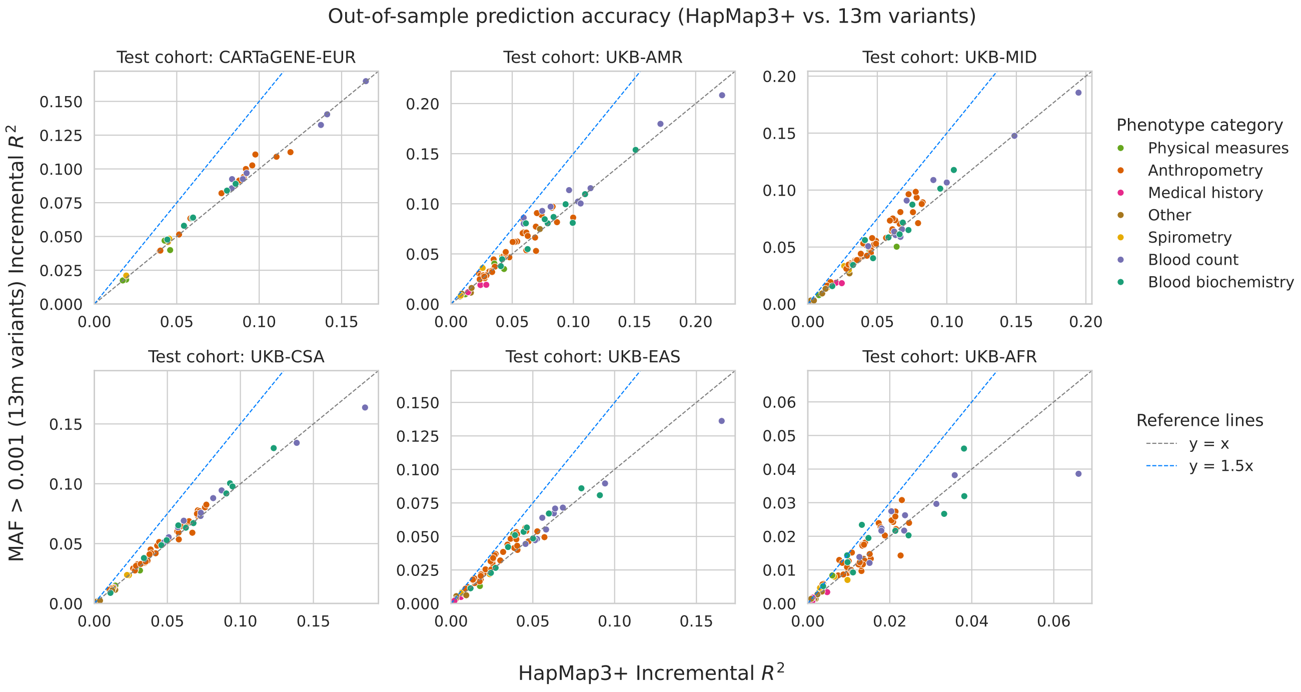

Figure S14: Systematic evaluation of PanUKB-derived PRS models across 75 continuous phenotypes and two variant sets. All polygenic scores were inferred from European GWAS summary statistics from the PanUKB initiative using `VIPRS v0.1`. The figure shows the comparative prediction accuracy between the HapMap3+ variant set on the x-axis (1.4 million variants) compared to the MAF>0.001 (13m) variant set on the y-axis. Each sub-panel compares the prediction accuracy for one of six held-out test cohorts across two biobanks: CARTaGENE and UK Biobank (UKB). The ancestry groups are EUR (European), AMR (Admixed American), MID (Middle Eastern), CSA (Central and South Asian), EAS (East Asian), and AFR (African). Colors denote different phenotype categories and dashed lines delineate the magnitude of the improvement in prediction accuracy.

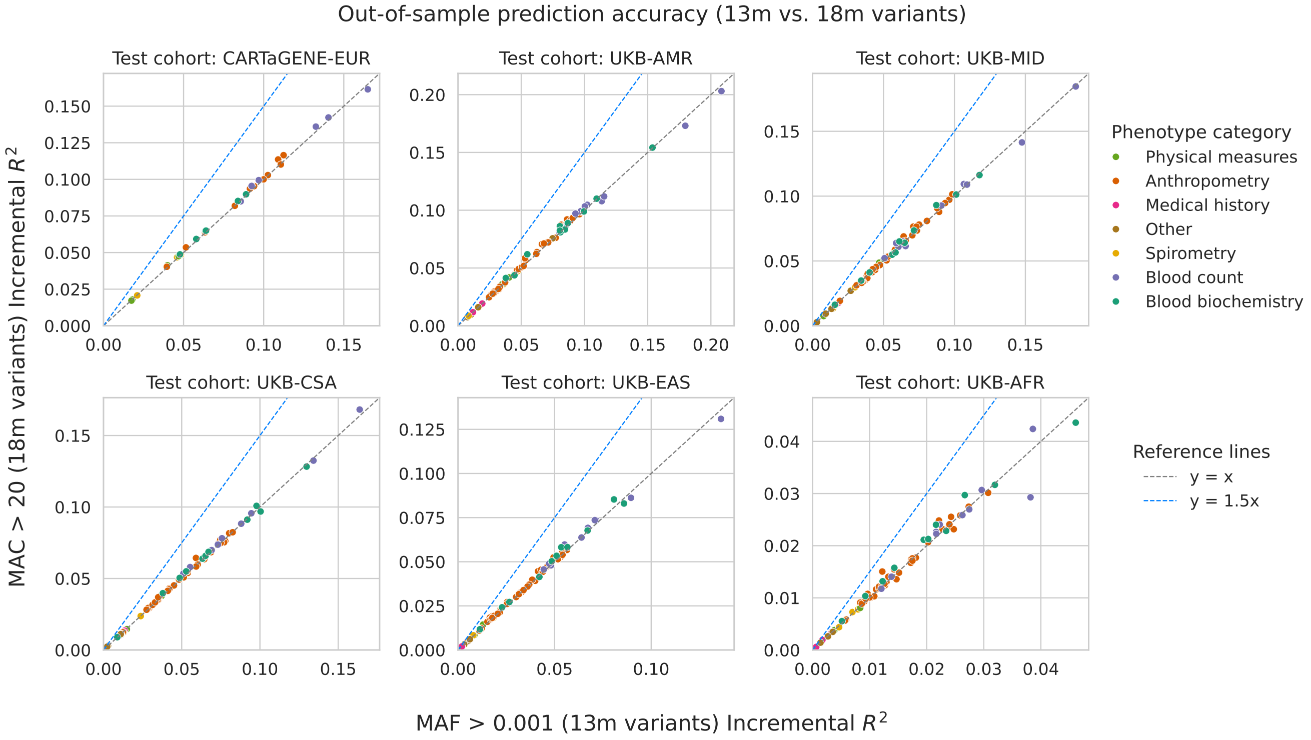

Figure S15: Systematic evaluation of PanUKB-derived PRS models across 75 continuous phenotypes and two variant sets. All polygenic scores were inferred from European GWAS summary statistics from the PanUKB initiative using `VIPRS v0.1`. The figure shows the comparative prediction accuracy between the MAF>0.001 (13m) variant set on the x-axis compared to the MAC>20 (18m) variant set on the y-axis. Each sub-panel compares the prediction accuracy for one of six held-out test cohorts across two biobanks: CARTaGENE and UK Biobank (UKB). The ancestry groups are EUR (European), AMR (Admixed American), MID (Middle Eastern), CSA (Central and South Asian), EAS (East Asian), and AFR (African). Colors denote different phenotype categories and dashed lines delineate the magnitude of the improvement in prediction accuracy.

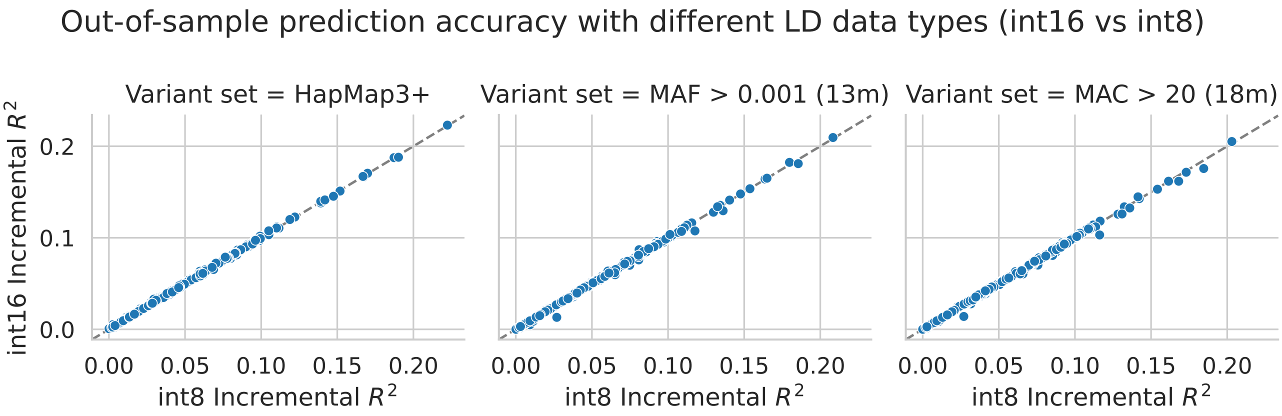

Figure S16: Comparison of held-out test prediction accuracy obtained when using LD matrices with `int8` (x-axis) vs. `int16` (y-axis) quantization across 75 phenotypes and six cohorts. All polygenic scores were inferred from European GWAS summary statistics from the PanUKB initiative using `VIPRS v0.1`. The held out cohorts include European samples from CARTaGENE as well as five non-European ancestry groups from the UK Biobank. This figure demonstrates that quantization to `int8` does not meaningfully affect prediction accuracy in most cases.

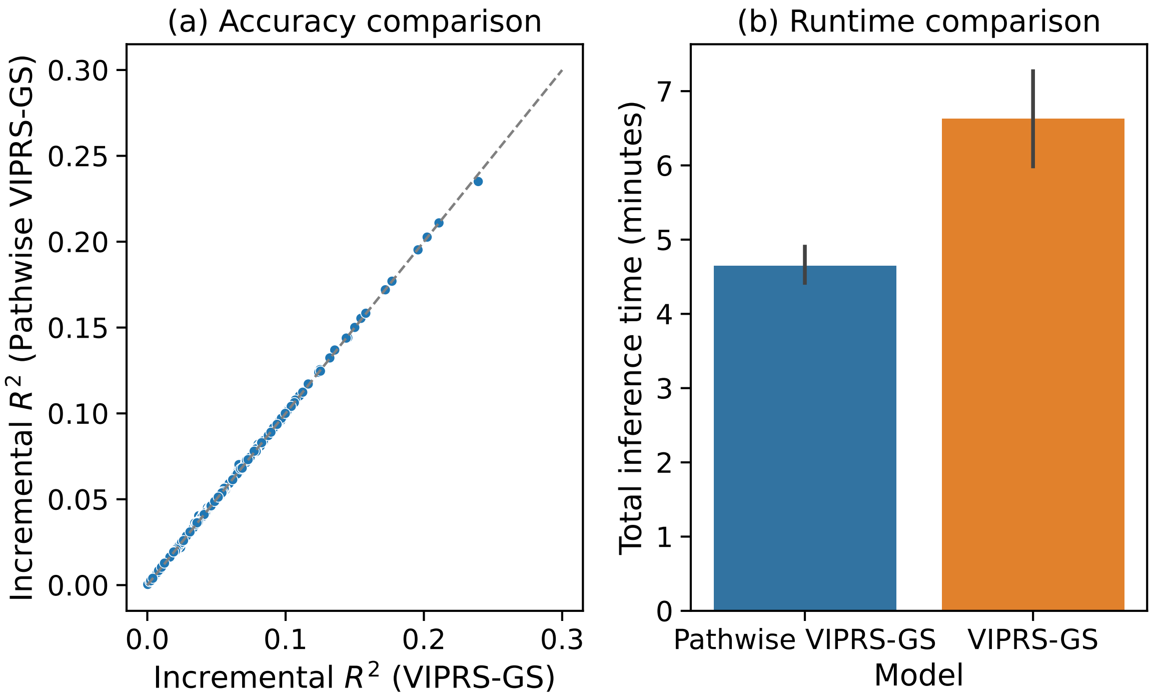

Figure S17: Comparison of the statistical and computational performance of the pathwise grid search algorithm against independent grid search on HapMap3+ variants. **(a)** compares the accuracy (incremental R^2) between regular grid search (x-axis) and pathwise grid search (y-axis) for all held-out cohorts and across 75 phenotypes in the Pan-UKB. **(b)** shows the total inference time (minutes) across all 22 chromosomes with the pathwise grid search algorithm (blue) vs. the independent grid search algorithm (orange).

S2 Supplementary Tables

Phenocode	Description	Category	LDSC h_2 (EUR)
48	Waist circumference	Anthropometry	0.223
49	Hip circumference	Anthropometry	0.250
50	Standing height	Anthropometry	0.588
51	Seated height	Anthropometry	0.291
20015	Sitting height	Anthropometry	0.449
21001	Body mass index (BMI)	Anthropometry	0.262
21002	Weight	Anthropometry	0.300
23098	Weight	Anthropometry	0.296
23099	Body fat percentage	Anthropometry	0.252
23100	Whole body fat mass	Anthropometry	0.275
23101	Whole body fat-free mass	Anthropometry	0.348
23102	Whole body water mass	Anthropometry	0.342
23104	Body mass index (BMI)	Anthropometry	0.268
23105	Basal metabolic rate	Anthropometry	0.336
23106	Impedance of whole body	Anthropometry	0.278
23107	Impedance of leg (right)	Anthropometry	0.253
23108	Impedance of leg (left)	Anthropometry	0.261
23109	Impedance of arm (right)	Anthropometry	0.268
23110	Impedance of arm (left)	Anthropometry	0.253
23111	Leg fat percentage (right)	Anthropometry	0.244
23112	Leg fat mass (right)	Anthropometry	0.255
23113	Leg fat-free mass (right)	Anthropometry	0.319
23114	Leg predicted mass (right)	Anthropometry	0.323
23115	Leg fat percentage (left)	Anthropometry	0.242
23116	Leg fat mass (left)	Anthropometry	0.256
23117	Leg fat-free mass (left)	Anthropometry	0.326
23118	Leg predicted mass (left)	Anthropometry	0.316
23119	Arm fat percentage (right)	Anthropometry	0.240
23120	Arm fat mass (right)	Anthropometry	0.247
23121	Arm fat-free mass (right)	Anthropometry	0.308
23122	Arm predicted mass (right)	Anthropometry	0.313
23123	Arm fat percentage (left)	Anthropometry	0.245
23124	Arm fat mass (left)	Anthropometry	0.255
23125	Arm fat-free mass (left)	Anthropometry	0.303
23126	Arm predicted mass (left)	Anthropometry	0.305
23127	Trunk fat percentage	Anthropometry	0.242
23128	Trunk fat mass	Anthropometry	0.265

23129	Trunk fat-free mass	Anthropometry	0.339
23130	Trunk predicted mass	Anthropometry	0.339
30600	Albumin	Blood biochemistry	0.145
30610	Alkaline phosphatase	Blood biochemistry	0.205
30620	Alanine aminotransferase	Blood biochemistry	0.124
30630	Apolipoprotein A	Blood biochemistry	0.182
30700	Creatinine	Blood biochemistry	0.213
30720	Cystatin C	Blood biochemistry	0.230
30730	Gamma glutamyltransferase	Blood biochemistry	0.180
30750	Glycated haemoglobin (HbA1c)	Blood biochemistry	0.210
30770	IGF-1	Blood biochemistry	0.252
30870	Triglycerides	Blood biochemistry	0.177
30890	Vitamin D	Blood biochemistry	0.071
30010	Red blood cell (erythrocyte) count	Blood count	0.214
30080	Platelet count	Blood count	0.282
30100	Mean platelet (thrombocyte) volume	Blood count	0.281
30140	Neutrophill count	Blood count	0.174
30180	Lymphocyte percentage	Blood count	0.157
30190	Monocyte percentage	Blood count	0.160
30200	Neutrophill percentage	Blood count	0.149
30250	Reticulocyte count	Blood count	0.208
30270	Mean sphered cell volume	Blood count	0.206
30300	High light scatter reticulocyte count	Blood count	0.225
135	Number of self-reported non-cancer illnesses	Medical history	0.069
2178	Overall health rating	Medical history	0.109
2217	Age started wearing glasses or contact lenses	Medical history	0.081
400	Time to complete round	Other	0.096
1180	Morning/evening person (chronotype)	Other	0.130
1239	Current tobacco smoking	Other	0.062
1717	Skin colour	Other	0.081
30530	Sodium in urine	Other	0.077
46	Hand grip strength (left)	Physical measures	0.122
47	Hand grip strength (right)	Physical measures	0.119
4080	Systolic blood pressure, automated reading	Physical measures	0.160
4194	Pulse rate	Physical measures	0.126
3062	Forced vital capacity (FVC)	Spirometry	0.223
3063	Forced expiratory volume in 1-second (FEV1)	Spirometry	0.205
3064	Peak expiratory flow (PEF)	Spirometry	0.112

Table S1: Pan-UK Biobank phenotypes analyzed in this study. The table includes the UKB phenotype code, phenotype description, general category, as well as LDSC heritability estimates in European samples (provided by Pan-UKB manifest [1]).

Ancestry group	Variant set	Number of variants	LD matrix storage size
AFR	HapMap3+	$1\,258\,340$	0.77 GB
AMR	HapMap3+	$1\,284\,038$	0.91 GB
CSA	HapMap3+	$1\,378\,881$	0.72 GB
EAS	HapMap3+	$1\,144\,401$	0.57 GB
MID	HapMap3+	$1\,305\,098$	0.88 GB
EUR	HapMap3+	$1\,431\,634$	0.63 GB
EUR	MAF $> 0.1\%$	$13\,482\,709$	37.68 GB
EUR	MAC > 20	$17\,708\,098$	59.54 GB

Table S2: LD matrix storage size for each ancestry group in the Pan-UKB. For European samples, we show LD matrix storage size across three different variant sets. LD matrices were estimated using banded masks with 3 centiMorgan window size. Entries of the matrix are stored using `int8` quantization.

References

[1] Konrad J. Karczewski, Rahul Gupta, Masahiro Kanai, Wenhan Lu, Kristin Tsuo, Ying Wang, Raymond K. Walters, Patrick Turley, Shawneequa Callier, Nikolas Baya, et al. "Pan-UK Biobank GWAS improves discovery, analysis of genetic architecture, and resolution into ancestry-enriched effects". In: *medRxiv* (2024). DOI: 10.1101/2024.03.13.24303864. eprint: https://www.medrxiv.org/content/early/2024/03/15/2024.03.13.24303864.full.pdf. URL: https://www.medrxiv.org/content/early/2024/03/15/2024.03.13.24303864.
